# NIR‐Triggered Crystal Phase Transformation of NiTi‐Layered Double Hydroxides Films for Localized Chemothermal Tumor Therapy

**DOI:** 10.1002/advs.201700782

**Published:** 2018-02-07

**Authors:** Donghui Wang, Naijian Ge, Tingting Yang, Feng Peng, Yuqin Qiao, Qianwen Li, Xuanyong Liu

**Affiliations:** ^1^ State Key Laboratory of High Performance Ceramics and Superfine Microstructure Shanghai Institute of Ceramics Chinese Academy of Sciences Shanghai 200050 China; ^2^ University of Chinese Academy of Sciences Beijing 100049 China; ^3^ Intervention Center Eastern Hepatobiliary Surgery Hospital the Second Military Medical University Shanghai 200438 China

**Keywords:** chemothermal therapy, crystal phase change, layered double hydroxides, near infrared

## Abstract

Construction of localized drug‐eluting systems with synergistic chemothermal tumor‐killing abilities is promising for biomedical implants directly contacting with tumor tissues. In this study, an intelligent and biocompatible drug‐loading platform, based on a gold nanorods‐modified butyrate‐inserted NiTi‐layered double hydroxides film (Au@LDH/B), is prepared on the surface of nitinol alloy. The prepared films function as drug‐loading “sponges,” which pump butyrate out under near‐infrared (NIR) irradiation and resorb drugs in water when the NIR laser is shut off. The stimuli‐responsive release of butyrate is verified to be related with the NIR‐triggered crystal phase transformation of Au@LDH/B. In vitro and in vivo studies reveal that the prepared films possess excellent biosafety and high efficiency in synergistic thermochemo tumor therapy, showing a promising application in the construction of localized stimuli‐responsive drug‐delivery systems.

## 1 Introduction

With shape memory effect, superelasticity, and biocompatibility, nitinol has been widely used in biomedical applications,^1^, ^2^, ^3^ especially tumor‐contacting implants and medical devices. One of the most commercially successful examples of utilization of nitinol is the production of expandable stents, which are applied in the palliation treatment of malignant obstruction of trachea, brochi, esophagus, and bile duct.^4^, ^5^, ^6^, ^7^, ^8^ The occlusion can be caused by many kinds of cancers, such as gallbladder carcinoma, esophagus carcinoma, cholangiocarcinoma, gastric carcinoma, and so on. However, tumor ingrowth and overgrowth into the nitinol stents often causes reocclusion, leading to durability reduction and function degradation subsequently.^9^ Therefore, developing nitinol stents with antitumor abilities is highly desirable.

A series of antitumor drug‐eluting stents (DESs) have been prepared by covering nitinol stents with drug‐loading polymer shells.^10^, ^11^, ^12^, ^13^ These DESs exhibited sustained antitumor drug release behavior and can be used for tumor chemotherapy. However, long‐term contact between tissues and polymer coatings usually leads to serious inflammatory responses.^14^ Besides, drug release amounts of the current DESs can hardly be controlled and will do harm to the nontargeted tissues, causing some unpleasant side effects. Near‐infrared (NIR)‐induced photothermal therapy is another treatment against tumor which has drawn tremendous attention due to its minimal invasiveness, spatiotemporal addressability, and high therapeutic efficiency. Some stents with photothermal effects have been designed and show preferable tumor inhibition effect.^15^ However, previous studies show that thermotherapy cannot eradicate tumor because of the inhomogeneous heat distribution within tumor tissues, resulting in inevitable tumor recurrence and metastasis.^16^, ^17^, ^18^ Efficient combination of chemotherapy and photothermal therapy can significantly increase the therapeutic effect compared to those sole treatments. A lot of drug delivery systems have been developed to realize combination chemotherapy and photothermal ablation. For instance, chemical drugs were directly loaded in photothermal nanoparticles, such as gold,^19^, ^20^, ^21^, ^22^ sulfide,^23^, ^24^, ^25^, ^26^ graphene and graphene‐like materials,^27^, ^28^, ^29^, ^30^ Prussian blue;^31^, ^32^ or loaded in mesoporous silica, smart polymer, aptamer, and cleavable linkages etc. modified on the photothermal particles.^33^, ^34^ However, most of the current reported chemothermal therapy platforms are prepared in the form of nanoparticles, which cannot be used in localized drug delivery systems. Construction of drug‐loading films with synergistic chemothermal tumor therapy still presents a considerable challenge.

**Figure 1:**
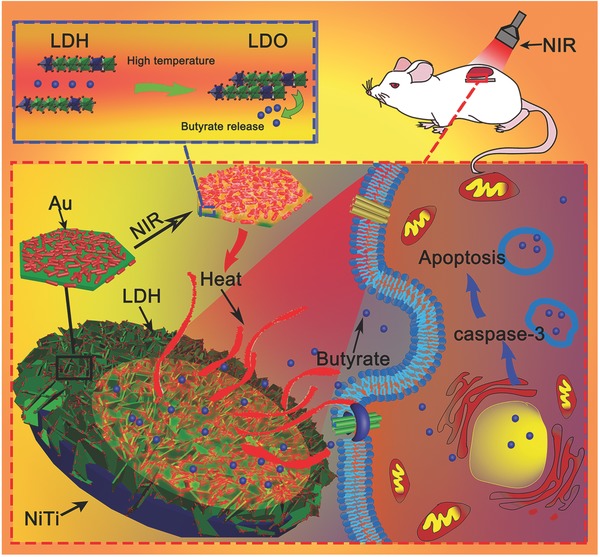
Illustration of the NIR‐triggered synergistic chemothermal tumor therapy of Au@LDH/B via phase transformation.

Recently, layered double hydroxides (LDHs) are widely investigated as drug carriers owning to their high biocompatibility and ion exchange capacities.^35^, ^36^ In addition, LDHs films can be easily grown on many kinds of biomedical metals, such as magnesium and its alloys, titanium and nitinol, making them suitable for surface drug‐loading layers construction on biomedical implants.^37^, ^38^, ^39^, ^40^ However, few studies focus on the temperature sensitivities of LDHs materials, which may be used in the preparation of stimuli‐responsive drug‐eluting platforms. In this study, we inserted butyrate, an agent with anticancer abilities, into the interlayer of NiTi‐LDHs constructed on the surface of nitinol via a simple hydrothermal treatment. The butyrate‐loaded LDHs films were further modified by gold nanorods (GNRs) with photothermal effect. As schematically shown in **Figure** **1**, the prepared film functions like a drug‐stored “sponge,” NIR can trigger the temperature increase of GNRs, leading to the crystal phase of the film change from layered double hydroxides to layered double oxides (LDOs), pushing the interlayer butyrate out. When the NIR irradiation is stopped, the crystal phase of the prepared film turns back to LDHs in water, excessive drugs in environment will be reabsorbed by the film accordingly. This newly designed drug‐loading film shows excellent biocompatibility and synergistic chemothermal therapy to tumors in vitro and in vivo, which we believe, will find a new avenue for potential applications in localized drug‐eluting systems.

## 2 Results and Discussions

### 2.1 Design and Characterization of Au@LDH/B

**Figure 2:**
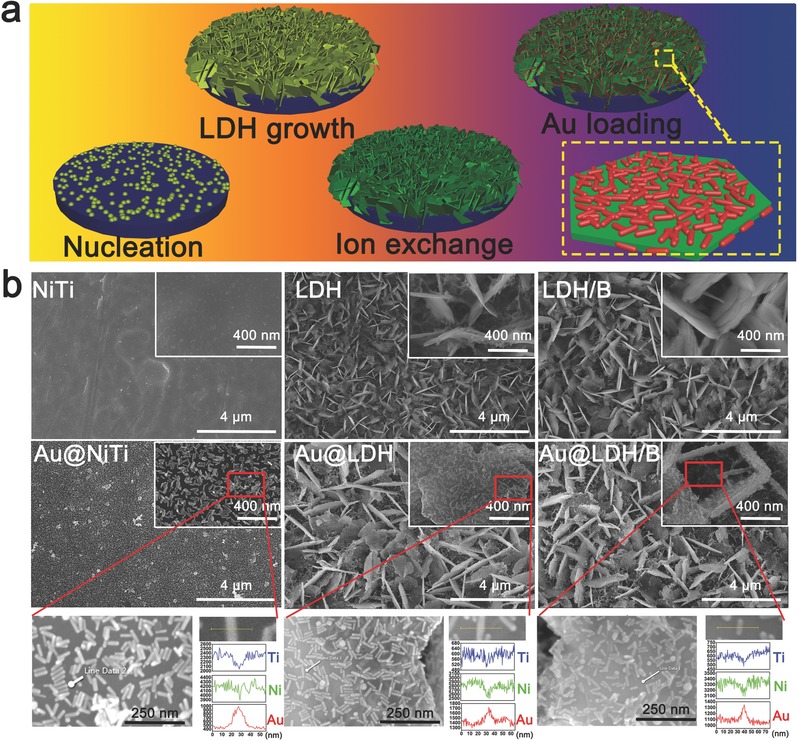
Preparation and characterization of Au@LDH/B. a) Schematic diagram depicting the formation of Au@LDH/B. b) SEM images of NiTi, LDH, LDH/B, Au@NiTi, Au@LDH, and Au@LDH/B samples; and the corresponding elemental line‐scanning spectra of Au@NiTi, Au@LDH, and Au@LDH/B samples.

**Figure** **2**a illustrates the general processes of engineering the NIR‐triggered butyrate‐eluting platform. We synthesized the butyrate‐inserted NiTi‐LDHs films (designated LDH/B) on nitinol substrates using a one‐step urea‐assisted hydrothermal treatment.^41^ Three steps (nucleation, crystal growth, and ion exchange) were involved in the formation of LDH/B. The details of each step are discussed in the Supporting Information. For comparison, a NiTi‐LDHs film without butyrate insertion was also constructed on the NiTi substrate and designated LDH. The prepared LDH and LDH/B showed similar platelet‐like structures. The width and length of the platelets were ≈2 µm, but their thickness was only ≈30 nm (Figure 2b). X‐ray diffraction (XRD) spectra of LDH and LDH/B confirmed their layered structure, and their interlayer distances were 0.762 and 0.783 nm, respectively (Figure S1, Supporting Information). Fourier transform infrared spectra verified the interlayer ions of LDH and LDH/B were carbonate and butyrate, respectively (Figure S2, Supporting Information).

GNRs were prepared through the previous reported seed‐mediated method.^42^, ^43^ The synthesized GNRs had an average diameter of 10 nm and length of 50 nm (Figure S3, Supporting Information). As evidenced by the scanning electron microscopy (SEM) images shown in Figure 2b, GNRs could be successfully immobilized on the surface of NiTi, LDH, and LDH/B, the GNRs modified NiTi samples were designated Au@NiTi, the GNRs modified LDH samples were designated Au@LDH, the GNRs modified LDH/B samples were designated Au@LDH/B. In addition, the corresponding elemental linear scanning spectra (Figure 2b) further confirmed the successful GNRs loading, consist with the results of X‐ray photoelectron spectra (Figures S4 and S5, Supporting Information). The loaded GNRs had similar coverage ratio on Au@NiTi, Au@LDH, and Au@LDH/B (≈50%, Figure S6, Supporting Information). However, owning to the higher specific surface area of Au@LDH and Au@LDH/B samples, they exhibited much higher GNRs loading ratio than Au@NiTi (75% vs 25%, Figure S7, Supporting Information).

**Figure 3:**
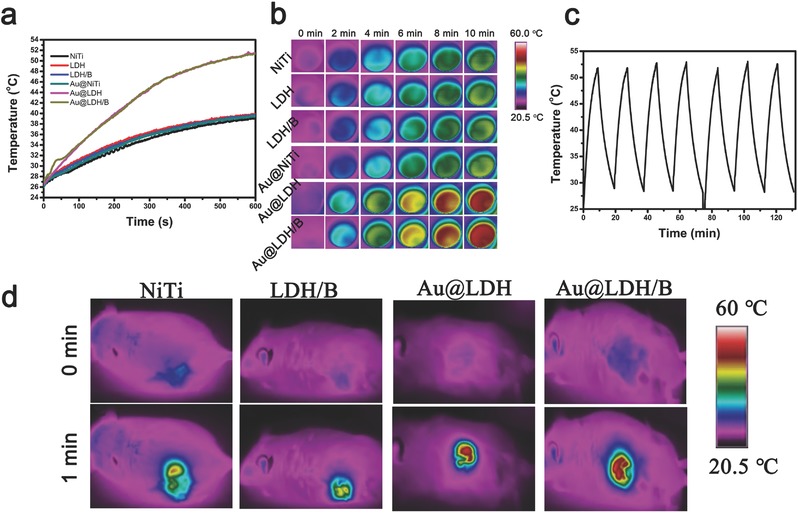
Photothermal effect of Au@LDH/B. a) Changes of surface temperature of different samples in water under NIR irradiation. b) Corresponding images showing the temperature distribution. c) Temperature variations of Au@LDH/B along with the off and on of NIR laser. d) Images showing the temperature changes of samples implanted in vivo.

To explore the photothermal conversion property, samples immersed in 500 µL ultrapure water were exposed to the 808 nm laser at a power density of 1.0 W cm^−2^ for 10 min. The surface temperature of Au@LDH and Au@LDH/B samples increased to 50 °C; while the temperature of Au@NiTi could only reach 38 °C, same as the samples without GNRs loading (**Figure** **3**a,b). When exposed to air, the surface temperature of Au@LDH and Au@LDH/B samples could even reach 67 °C after 5 min NIR irradiation (0.5 W cm^−2^) (Figure S8, Supporting Information). Besides, the GNRs‐loading samples exhibited remarkable photostability under NIR irradiation for seven cycles (Figure 3c). Such great photothermal conversion property could be kept in vivo. After implanted subcutaneously in mice, temperature of Au@LDH and Au@LDH/B samples rose to 48 °C in 1 min under NIR irradiation, 10 °C higher than the samples without GNRs modification (Figure 3d and Figure S9, Supporting Information). The photothermal conversion efficiency η of the GNRs‐modified NiTi‐LDHs film was calculated to be 21.7% (Equations S4–S13, Figures S10 and S11, Supporting Information), comparable to free GNRs.^44^ The relatively high photostability and photothermal conversion efficiency indicate that Au@LDH and Au@LDH/B samples possess excellent photothermal conversion properties, thus can be used as localized photothermal conversion platforms for the potential application in treatment against tumor.

### 2.2 NIR Triggered Crystal Phase Transformation of Au@LDH/B

**Figure 4:**
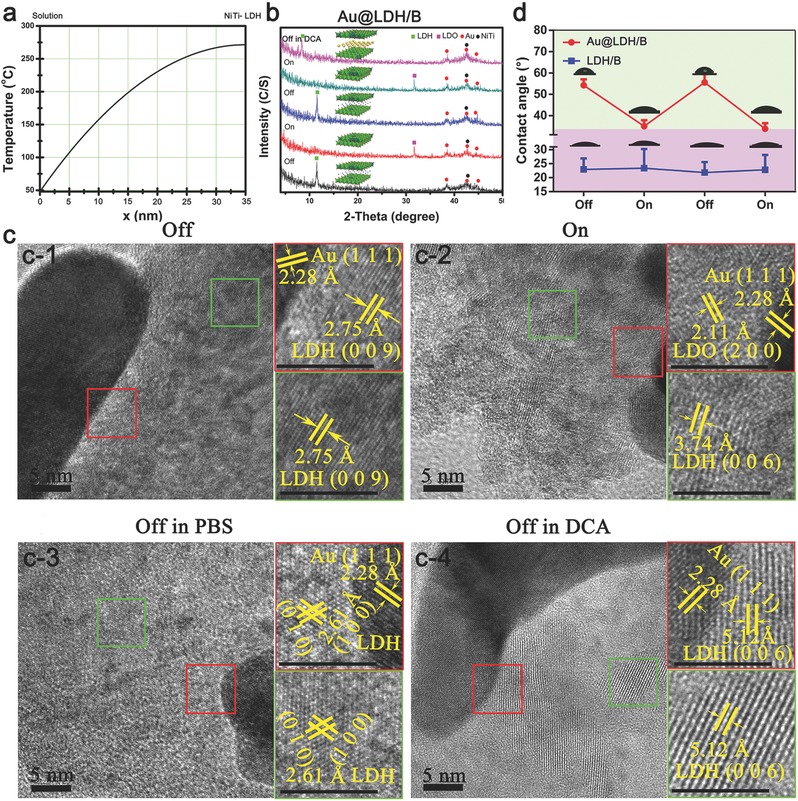
Crystal phase changes of Au@LDH/B under NIR. a) Calculated temperature distribution of GNRs immobilized on NiTi‐LDHs films. b) XRD patterns of Au@LDH/B before and after NIR irradiation, and the XRD patterns of the NIR‐irradiated Au@LDH/B being immersed in PBS or DCA solution. c) TEM images of Au@LDH/B before (c‐1) and after (c‐2) NIR irradiation, and the TEM images of the NIR‐irradiated Au@LDH/B being immersed in PBS (c‐3) or DCA solution (c‐4). d) Water contact angles of Au@LDH/B and LDH/B before and after NIR irradiation.

Different from free GNRs, the GNRs immobilized on NiTi‐LDHs films are exposed to an anisotropic environment, leading to an inhomogeneous temperature distribution. Heat can be hardly dissipated in the contacting area between GNRs and NiTi‐LDHs films, leading to the temperature of the contact area is much higher than the area exposed to water. The temperature distribution of GNRs immobilized on NiTi‐LDHs films was thus calculated based on a model shown in Figure S12, Supporting Information. The detailed calculation can be found in Equations S14–S24 (Supporting Information), and the result is presented as Equation S25, Supporting Information, the corresponding function plots is shown in **Figure** **4**a. It can be found that the temperature of the contact area of GNRs and LDHs films could reach as high as 271 °C, which surpassed the onset temperature of crystal phase transformation of NiTi‐ LDHs (251 °C, Figure S13, Supporting Information). XRD patterns of Au@LDH/B samples before and after NIR irradiation in air are shown in Figure 4b. Before NIR irradiation, characteristic peak centered ≈11°, corresponding to the (0 0 3) crystal face of NiTi‐LDHs,^45^, ^46^ was detected. After NIR irradiation, the peaks corresponding to NiTi‐LDHs crystal phase vanished, while a peak corresponding to (1 0 1) crystal face of NiTi‐LDOs appeared at 32°,^47^ indicating the phase transformation of Au@LDH/B under NIR irradiation. However, NiTi‐LDOs are not stable and will easily revert to NiTi‐LDHs by reacting with water.^48^ Besides, NiTi‐LDOs have no interlayer anions, they need absorb anions in the environment to achieve the reverse phase transformation, so the interlayer anions of the newly formed NiTi‐LDHs are determined by the types of anions in the environment. XRD tests verified the reserve phase transformation of Au@LDH/B samples. As shown in Figure 4b, the crystal phase of the irradiated Au@LDH/B turned back to NiTi‐LDHs after being immersed in phosphate buffered saline (PBS) for 30 min; immersing the NIR‐irradiated Au@LDH/B sample into dodecanoic (DCA) sodium solution also resulted the regeneration of NiTi‐LDHs, but their (0 0 3) peaks moved from 11.3° to 8.5°, indicating the large‐sized DCA ions had entered into the interlayer of NiTi‐LDHs.

The high‐resolution transmission electron microscopy (TEM) images (Figure 4c) and the corresponding energy dispersive spectrometer (EDS) spectra (Figure S14, Supporting Information) of Au@LDH/B before (Figure 4c‐1) and after (Figure 4c‐2) the NIR irradiation and further immersed in PBS (Figure 4c‐3) or DCA solution (Figure 4c‐4) are consistent with the XRD results. Before NIR irradiation, only characteristic fringes corresponding to NiTi‐LDHs could be found, no matter in the area near (red square) or away from (green square) the modified GNRs. After NIR irradiation, the crystal phase of the area near GNRs changed to NiTi‐LDOs, while fringes corresponding to NiTi‐LDHs could still be observed away from GNRs. Immerse the NIR‐irradiated Au@LDH/B into PBS, characteristic fringes corresponding to NiTi‐LDOs disappeared, only lattices of NiTi‐LDHs could be found. Immersing the NIR‐irradiated Au@LDH/B into DCA solution would also lead to the formation of NiTi‐LDHs, but the detected (0 0 6) lattice became larger, indicating DCA ions had entered into the interlayer of NiTi‐LDHs, confirming the interlayer ions of the regenerated LDHs were determined by the anions in the environment. Therefore, the interlayer drugs of the prepared films are exchangeable, making them promising in the high‐throughput antitumor drugs screening.

When Au@LDH/B samples were immersed in water, their crystal phase could also change along with the off and on of NIR laser (Figure S15a, Supporting Information). What's more, the phase change abilities of Au@LDH/B could be maintained even after 20 phase change cycles (Figure S15b, Supporting Information), indicating the high phase change stabilities of Au@LDH/B samples. LDH/B samples have no photothermal conversion capacities, so their crystal phase did not change along with NIR irradiation (Figure S16, Supporting Information).

As discussed above, NiTi‐LDOs can react with water dropped on their surfaces, resulting in their higher hydrophilicity than NiTi‐LDHs. NIR irradiation can change the crystal phase of Au@LDH/B but has no effect to LDH/B, making NIR laser can be used to control the water contact angles of Au@LDH/B but have no influence on that of LDH/B (Figure 4d). The water contact angle tests further confirmed the phase changes of Au@LDH/B before and after NIR irradiation.

### 2.3 NIR‐Triggered Butyrate Release of Au@LDH/B

**Figure 5:**
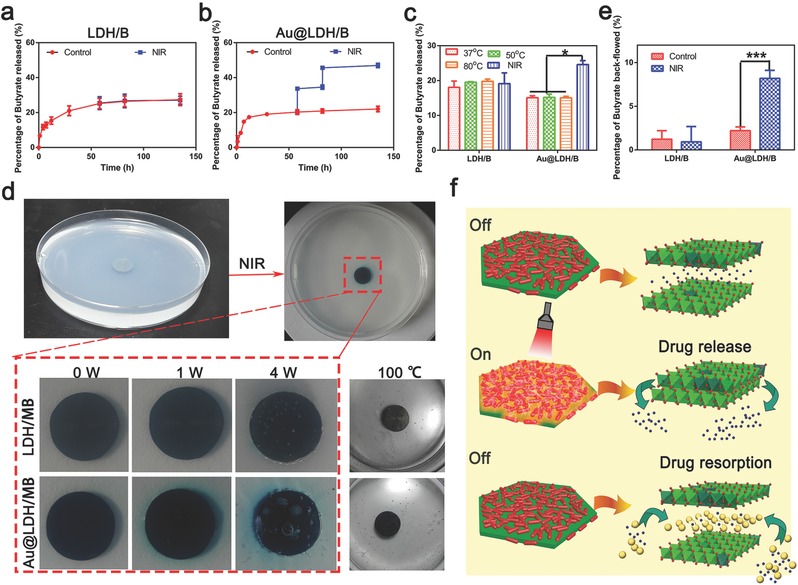
NIR controlled butyrate release of Au@LDH/B. a) Butyrate release of LDH/B before and after NIR irradiation. b) Butyrate release of Au@LDH/B before and after NIR irradiation. c) Butyrate release of LDH/B and Au@LDH/B immersing in PBS for 4 h at different temperatures or expose to NIR irradiation. d) Images showing the MB release from LDH/B and Au@LDH/B immobilized in agar plate in different conditions. e) Butyrate resorption abilities of LDH/B and Au@LDH/B before and after NIR irradiation. f) Illustration of the mechanism of the interlayer ions release and resorption of Au@LDH/B sample.

Butyrate release of LDH/B and Au@LDH/B samples before and after NIR irradiation were investigated. Results are presented in **Figure** **5**a,b. NIR irradiation had no influence on the butyrate release of LDH/B, but significantly increased the butyrate release amounts of Au@LDH/B. To explore whether it was the high environmental temperature of Au@LDH/B under NIR irradiation induced its burst butyrate release, we immersed LDH/B and Au@LDH/B samples in PBS with different temperatures, their butyrate release amounts were examined 4 h later. Even up to 80 °C, much higher than the attainable temperature via NIR irradiation, the butyrate release amounts of both samples still showed little differences compared to the samples immersed in PBS at 37 °C, indicating environmental temperature had little influences on samples' butyrate release (Figure 5c).

To make the NIR‐responsive interlayer ions release of LDH/B and Au@LDH/B visualization, their interlayer butyrate ions were replaced by methyl blue (MB), a coloring agent. The obtained samples, denoted as LDH/MB and Au@LDH/MB, were then immobilized in an agar plate and irradiated by NIR with different power density. As shown in Figure 5d, when irradiated by NIR with a power density of 1 W cm^−2^, a blue circle appeared around Au@LDH/MB sample, indicating the release of MB. The blue circle became larger when the power density of NIR rose to 4 W cm^−2^ and a lot of bubbles appeared on the sample surface, manifesting the ultrahigh surface temperature of Au@LDH/B after NIR irradiation. On the contrary, no blue circle could be detected around LDH/MB sample after NIR irradiation. Place the agar plates into a 100 °C oven, many bubbles appeared around both samples, but no blue circle appeared, further confirmed that environmental temperature had little influences on the release amounts of interlayer anions of NiTi‐LDHs films.

As illustrated in Figure 5f, the NIR‐responsive butyrate release of Au@LDH/B is related to its NIR‐responsive phase change. When NiTi‐LDHs transform to NiTi‐LDOs, their interlayer distance decreases, anions stored in the interlayer thus being pumped out. Stop NIR irradiation, environmental anions will flow back to the interlayer to achieve the reverse phase transformation. Therefore, the NIR‐irradiated Au@LDH/B had a relatively high ability to resorb butyrate when it was immersed in a butyrate solution (Figure 5e). The drug‐resorbing abilities of Au@LDH/B guarantee not too many drugs being left in the environment after NIR irradiation, which may endow Au@LDH/B with high biosafety.

### 2.4 In Vitro and In Vivo Evaluation of the Tumor Inhibition Abilities of Au@LDH/B

Considering its great photothermal conversion properties and NIR‐triggered butyrate release abilities, the in vitro tumor therapeutic efficiency of Au@LDH/B was investigated in detail. No significant cytotoxicity of the investigated samples to normal cells (HIBEpiC) could be observed (Figure S17, Supporting Information). Live/dead stained images (Figure S18, Supporting Information), early apoptosis ratio (Figure S19, Supporting Information), and apoptosis related genes expressions (Figure S20, Supporting Information) of normal cells cultured on different samples showed little differences, further confirming the good biocompatibility of the prepared films.

**Figure 6:**
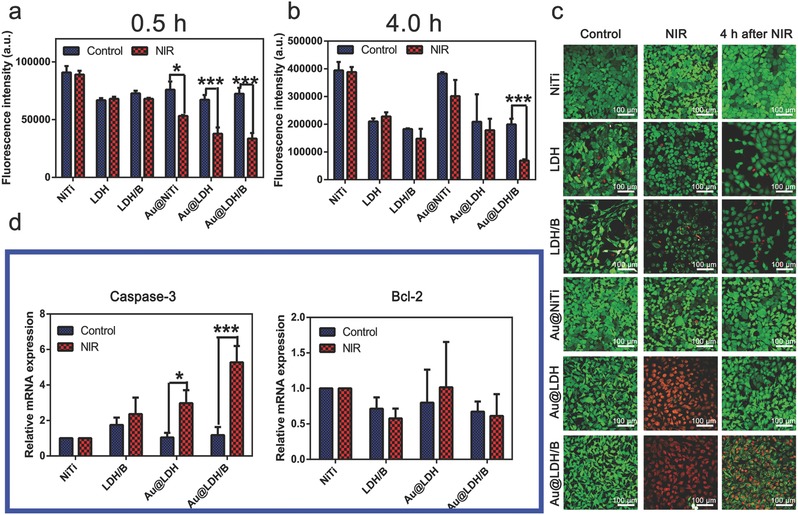
In vitro evaluation of the tumor inhibition abilities of Au@LDH/B. a) Viability of cancer cells cultured on different samples just after NIR irradiation, cells cultured on samples without NIR irradiation were set as control. b) Viability of cancer cells cultured on different samples 4 h after the NIR irradiation, cells cultured on samples without NIR irradiation were set as control. c) CLSM images of the live/dead stained cancer cells cultured on different samples before (control), just after, and 4 h after NIR irradiation. d) Relative expression of the apoptosis related genes caspase‐3 and Bcl‐2 in cancer cells cultured on different samples before (control) and after NIR irradiation.

The viabilities of cancer cells (RBE) cultured on different samples were assayed by alarm blue tests immediately or 4 h after the NIR irradiation, results are presented in **Figure** **6**a,b, respectively. The viabilities of cancer cells cultured on the GNRs loaded samples (Au@NiTi, Au@LDH, and Au@LDH/B) obviously decreased after the NIR treatment, which was due to their photothermal effect. Four hours after the NIR treatment, viabilities of cancer cells cultured on Au@NiTi and Au@LDH had recovered, but cells on Au@LDH/B were still inhibited. Confocal laser scan microscopy (CLSM) images of live/dead stained cancer cells cultured on different samples are presented in Figure 6c. Cancer cells survived (green) on all samples without NIR irradiation. After NIR treatment, cells on Au@LDH and Au@LDH/B turned red, indicating cells were in a dying state. However, 4 h after the NIR treatment, cancer cells cultured on Au@LDH turned back to green; while red dye had totally entered into the nucleus of cancer cells cultured on Au@LDH/B, indicating the death of cells on Au@LDH/B (Figure S21, Supporting Information). SEM images of RBE cells cultured on different samples before and after NIR irradiation further confirmed that Au@LDH/B possessed a lasting tumor killing ability; while Au@LDH could only inhibit cancer cells during the NIR treatment (Figure S22, Supporting Information).

Cells mainly die through apoptosis; the early apoptosis of cells was detected by a JC‐1 assay (Figure S23, Supporting Information). In this test, cells are stained to green or red based on their apoptosis states. A strong green fluorescence intensity means cells are in an early apoptosis state, while red fluorescence can be seen when cells are in a survival state. The early apoptosis rate can be represented by the ratio between the fluorescence intensities of green and red fluorescence. NIR treatment showed little adverse effect on the early apoptosis of cancer cells cultured on NiTi, LDH, LDH/B, and Au@NiTi, but effectively improved the early apoptosis rate of cancer cells cultured on Au@LDH and Au@LDH/B. Four hours after NIR treatment, the early apoptosis rate of cancer cells on Au@LDH decreased, while cancer cells on Au@LDH/B still presented a high early apoptosis rate.

The expressions of the apoptosis‐related genes caspase‐3 and Bcl‐2 of cancer cells cultured on different samples before and after NIR irradiation were evaluated by real‐time polymerase chain reaction. The results are presented in Figure 6d. A high expression of the proapoptotic gene in cancer cells cultured on Au@LDH was observed after NIR irradiation, cells on the NIR‐irradiated Au@LDH/B samples presented an even higher caspase‐3 expression; while the expression of the antiapoptotic gene Bcl‐2 showed little differences in cells cultured on different samples before and after NIR treatment.

Au@LDH samples only provide thermal therapy, their surface temperature increase under NIR irradiation, leading to the thermal damages to cancer cells. However, cancer cells can survive from hyperthermia and retain their viability through autophagy,^49^ their activities will recover soon after the NIR laser being shut off. Au@LDH samples thus possess no lasting antitumor effect. Except for high surface temperature, Au@LDH/B samples will release butyrate, an antitumor agent, under NIR irradiation, providing a distinct synergistic effect of chemothermal therapy, resulting in its high efficiency in RBE cells killing. Other kinds of cancer cells (MG63, 4T1) could also be effectively killed by Au@LDH/B under NIR irradiation (Figures S24 and S25, Supporting Information).

**Figure 7:**
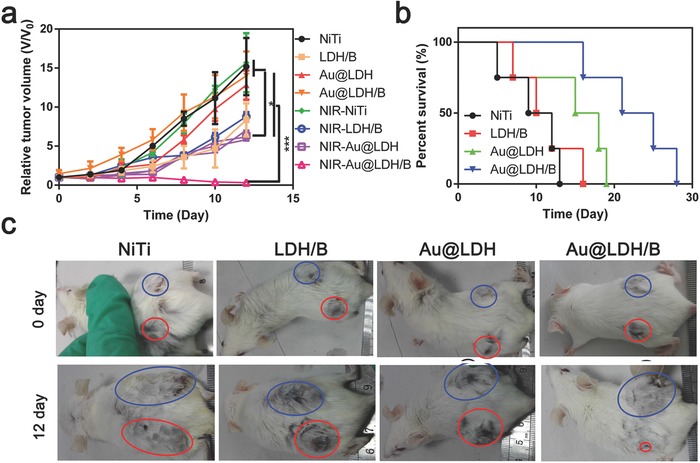
In vivo evaluation of the tumor inhibition abilities of Au@LDH/B. a) Relative tumor volume of mice implanted with different samples with and without NIR treatment. b) Survival rate of mice implanted with different samples. c) Representative digital images of tumor‐bearing mice after being implanted with different samples for various time periods, the left tumor was irradiated by NIR (red circle), the right tumor was set as control (blue circle).

Encouraged by the outstanding in vitro anticancer effects, the in vitro therapeutic efficiency of Au@LDH/B samples was further evaluated. Balb/c mice were used in the tumor model developing. Two tumors were planted on the back of each mouse, the left tumors were irradiated by NIR with a power density of 1 W cm^−2^ for 10 min every day, and the right tumor was set as control. The tumor‐bearing mice were divided into four groups, NiTi, LDH/B, Au@LDH, and Au@LDH/B plates with a diameter of 6 mm and thickness of 1 mm were implanted under each tumor. Without NIR irradiation, compared to the NiTi group, only the LDH/B group showed inhibited tumor growth (**Figure** **7**a), attributing to its butyrate release. Au@LDH and Au@LDH/B samples showed no tumor growth inhibition effect, indicating their low toxicity. However, after NIR irradiation, Au@LDH samples presented a similar tumor inhibitory rate as LDH/B samples owning to their photothermal effect; benefiting from the synergistic chemo‐ and photothermal therapy, the growth of tumor on mice implanted with Au@LDH/B samples had been completely inhibited after NIR irradiation, which can be visually confirmed by comparing the tumors sizes in various time durations, as shown in the representative digital photos (Figure 7c and Figure S26, Supporting Information). Besides, the Au@LDH/B implanted mice showed the longest survival time (Figure 7b) and highest activity (Movies S1–S4, Supporting Information) among mice implanted with different samples, verifying the effectiveness of Au@LDH/B in cancer therapy under NIR irradiation.

Tumors were taken out 12 d after the sample implantation for hematoxylin and eosin (H&E) staining (Figure S27, Supporting Information). A compact distribution of tumor cells could be observed in tumor tissue contacting NiTi, Au@LDH, and Au@LDH/B, while tumor cells contacting LDH/B were relatively sparsely distributed. NIR irradiation had no adverse effect on tumor tissue contacting NiTi and LDH/B but could effectively reduce cell number in tumor tissues contacting Au@LDH and Au@LDH/B. Notably, in the Au@LDH/B group most of the tumor cell nucleus had fractured into small debris after NIR irradiation, indicating the apoptosis of tumor cells. Liver, kidney, lung, spleen, and heart were also taken out for H&E staining (Figure S28, Supporting Information). Serious metastasis in liver (pointed by black arrows in Figure S28, Supporting Information) could be found in mice implanted with NiTi and Au@LDH, while no significant histopathological changes could be observed in mice implanted with LDH/B and Au@LDH/B, indicating LDH/B and Au@LDH/B samples possess no systemic toxicity to normal tissues and have tumor metastasis inhibition abilities, which may benefit from their butyrate release. In addition, the body weights of the mice in different groups did not have any noticeable changes during the experiments (Figure S29, Supporting Information), demonstrating satisfactory in vivo biocompatibility and biosafety, attributing to the drug‐resorbed abilities of the prepared films.

## 3 Conclusions

In summary, an intelligent localized drug‐loading film based on GNRs‐modified NiTi‐LDHs was designed for the synergistic chemothermal tumor therapy. The prepared film works like a “sponge,” it absorbs and stores drugs inside its crystal lattice, and pump drugs outside when the film is “squeezed” by NIR; when the NIR irradiation is stopped, the excess drugs in the environment can be reabsorbed by the drug‐loading “sponge,” endow the prepared films with high biocompatibility. The NIR‐controlled drug release is benefited from the phase transformation of NiTi‐LDHs. When Au@LDH/B exposes to NIR, the photothermal effect of GNRs increase the temperature of NiTi‐LDHs film, leading to its crystal phase change from layered double hydroxides to layered double oxides, butyrate ions stored interlayer release accordingly. In vitro and in vivo experiments verified Au@LDH/B samples present distinct synergistic effect in the chemothermal tumor therapy, which is promising to overcome the inevitable tumor recurrence and metastasis resulted from the inhomogeneous ablation of single thermal therapy. Therefore, the newly designed GNRs‐modified butyrate‐inserted NiTi‐LDHs films show great potential to be used in the surface modification of implants in contact with tumor tissues.

## Conflict of Interest

The authors declare no conflict of interest.

## Supporting information

Supplementary

Supplementary

Supplementary

Supplementary

Supplementary
