# Supplementary material for: NIR‐Triggered Crystal Phase Transformation of NiTi‐Layered Double Hydroxides Films for Localized Chemothermal Tumor Therapy
Source: Adv Sci (Weinh). 2018 Feb 7;5(4):1700782. doi: 10.1002/advs.201700782 (PMC5908485; doi:10.1002/advs.201700782)
Supplement: Supplementary file 1 — Supplementary [file ADVS-5-1700782-s002.pdf]

Copyright WILEY-VCH Verlag GmbH & Co. KGaA, 69469 Weinheim, Germany, 2018.

ADVANCED SCIENCE Open Access

Supporting Information

for *Adv. Sci.,* DOI: 10.1002/advs.201700782

NIR-Triggered Crystal Phase Transformation of NiTi-Layered Double Hydroxides Films for Localized Chemothermal Tumor Therapy

*Donghui Wang, Naijian Ge, Tingting Yang, Feng Peng, Yuqin Qiao, Qianwen Li, and Xuanyong Liu**

Copyright WILEY-VCH Verlag GmbH & Co. KGaA, 69469 Weinheim, Germany, 2016.

Supporting Information

NIR-Triggered Crystal Phase Transformation of NiTi-Layered Double Hydroxides Films for Localized Chemo-Thermal Tumor Therapy

*Donghui Wang, Naijian Ge, Tingting Yang, Feng Peng, Yuqin Qiao, Qianwen Li and Xuanyong Liu**

Experimental section

Preparation of Gold Nanorods: Cetyltrimethyl ammonium bromide (CTAB, 0.1 M, 5 mL, Aladdin, China), $HAuCl_4$ solution (0.02 M, 0.12 mL, Aladdin, China) and distilled water (2.5 mL) were mixed under magnetic stirring. After obtaining a yellow solution, iced $NaBH_4$ solution (0.01 M, 0.6 mL, Aladdin, China) was dropped into the mixture, continued to stir for a further 2 min and aged for 4 h at 27 ~ 30, the gold nanorods (GNRs) crystal seeds solution could be obtained. CTAB solution (0.1 M, 30 mL), $AgNO_3$ solution (0.01 M, 0.75 mL, Aladdin, China) and $HAuCl_4$ solution (0.02 M, 1.5 mL) were mixed in a 50 mL conical flask under magnetic stirring and a red solution could be obtained. Ascorbic acid (Aladdin, China) was used to titrate the red solution to colorless, continued to stir for a further 2 min and the GNRs growth solution could be formed. Added seeds solution (72 μL) into the growth solution and stirred for 10 s. After 12 hours' standing, the GNRs sol could be prepared.

Preparation of NiTi-LDHs and Butyrate-Inserted NiTi-LDHs Films: The NiTi-LDHs films were directly growth on the surface of nitinol via hydrothermal treatment. Briefly, a commercially available nitinol (50.8 at.% Ni) stick with a diameter of 12 mm was cut into plates with a thickness of 1 mm. The nitinol plates were thoroughly cleaned in water and ethanol by ultrasonic treatment. The precursor solution of NiTi-LDHs were prepared by mixing $NiCl_2.6H_2O$ (1.43 g, Sinopharm Chemical Reagent, China), $TiCl_4$ (0.25 mL, Sinopharm Chemical Reagent, China), HCl (0.25 mL, 36%-38%, Sinopharm Chemical Reagent, China), urea (6.5 g, Aladdin, China) with ultrapure water (1000 mL). Then the nitinol plates were placed into a 100 ml Teflon-lined stainless vessel, and the precursor solution (35 mL) was poured into the vessel and sealed. The vessel was put into a convective oven with the temperature pre-heated to 120 °C. After an elapsed time of 24 h, the vessel was taken out washed with copious of ultrapure water, boiled in water for 1 h and dried in air. To prepare butyrate inserted NiTi-LDHs films, sodium butyrate (33 g, Aladdin, China) was added into the precursor solution. The other treatment conditions were the same as the preparation processes of LDH sample, and the resulted specimens were designated as LDH/B.

Gold Nanorods Loading: NiTi, LDH and LDH/B samples were vertically placed into 4-mL tubes, 3-aminopropyltrimetyoxysilane solution (APS, 3 v%, 2 mL, Sinopharm Chemical Reagent, China) was added into each tube and the sample-containing tube was ultrasonically treated for 2 h. Then samples were cleaned by copious ultrapure water and immersed in a NaCl solution (6 mM, 1.5 mL) containing poly(styrene sulfonate) (PSS, 0.1 g/mL, Aladdin, China), placed on a shaker for 24 h at 37 ℃. GNRs were collected by centrifuging and redispersed in NaCl solution (100 mM). Samples were immersed into the GNRs contained solution (1.2 mL for each sample) for 48 h at 37 ℃. The GNRs loaded NiTi, LDH and LDH/B samples were denoted as Au@NiTi, Au@LDH and Au@LDH/B respectively.

Sample Characterization: The prepared specimens were characterized by scanning electron microscopy (SEM; S-4800, Hitachi, Japan), transmission electron microscopy (TEM, Tecnai G2 F20, Japan), energy dispersive spectrometer (EDS, JAX-8100, Japan), X-ray diffraction (XRD; Rigaku, Japan), fourier transform infrared spectroscopy (FTIR; FTIR-7600, Lambda Scientific, Australia), X-ray photoelectron spectroscopy (XPS; RBD upgraded PHI-5000C ESCA system, USA). Samples were directly characterized by all of the instruments above directly except for TEM an FTIR, in which powders were scraped from the samples for better characterization. Besides, the surface wettability of samples was measured by contact angle measurement (Automatic Contact Angle Meter Model SL200B, Solon information technology Co., Ltd, China). Thermogravimetric curve was tested using a simultaneous thermal analyzer (STA 409PC, Netzsch, Germany) with a heating rate of 10 ℃/min.

Photothermal Effect Characterization: Samples were immersed in 1 mL phosphate buffered saline (PBS, Hyclone, USA) and exposed to 808 nm laser irradiation (1 W/cm^2) for

10 min, an infrared thermal imager (FLIR A325sc, USA) was used to record the temperature changes of different samples. The temperature changes of samples exposed to air under NIR irradiation (0.5 W/cm^2) were also tested.

Interlayer Drugs Release Detection: Samples were immersed in 1 mL PBS at 37 °C without stirring for various periods of time. The amounts of released butyrate of different samples before and after NIR irradiation (1 W/cm^2, 10 min) were determined by analyzing the resulting solutions using ultraviolet and visible spectrophotometry (UV−Vis; Lambada 750, PerkinElmer, USA) at a absorbance wavelength of 201 nm. The butyrate release amounts of samples immersed in PBS with different temperature (37 ℃, 60 ℃ and 80 ℃) for 4 h were also characterized.

To visualize the interlayer ions release of NiTi-LDHs under NIR irradiation, methyl blue (MB, Aladdin, China) was inserted into the interlayer of NiTi-LDHs follow the method described in the preparation of LDH/B sample. The MB inserted NiTi-LDHs sample was denoted as LDH/MB. GNRs were subsequently loaded on the surface of LDH/MB and the obtained samples were denoted as Au@LDH/MB. LDH/MB and Au@LDH/MB samples were immobilized in agar plates and exposed to NIR irradiation with different power density (1 W/cm^2 and 4 W/cm^2) for 10 min. As MB is a strong coloring agent, the MB release can be easily observed with naked eyes.

Cell Culture: The human cholangiocarcinoma cell line RBE, osteosarcoma cell line MG63 and murine breast tumor cell line 4T1 were purchased from Cell Bank of Chinese Academy of Science. The human intrahepatic biliary epithelial cells (HIBEpic) were obtained from Sciencell (USA). All of the cells were maintained in the media provided by suppliers in a humidified atmosphere of 5% CO_2 at 37 °C. Based on the cell conditions, cells were passaged at a ratio of 1:2−1:4 every 2−4 days.

Cell Viability: The proliferation rate of cells cultured on different samples were measured by the alamarBlue™ assay (AbD Serotec Ltd., UK). The early apoptosis of cells cultured on different samples were tested by a JC-1 kit (Beyotime, China). In both of the above tests, cells were seeded on the specimen surfaces with a density of 5 × 10^4 cells/well and cultured for 4 days. Before the above detection, samples were divided into two groups, one group was irradiated by NIR (1 W/cm^2) for 10 min, the other group was set as control.

Live/Dead Cell Staining: Cells were seeded on the specimens with a density of 5 × 10^4 cells/well and cultured for 4 days. Then the cells were stained by a live/dead cell staining kit (Biovaision, USA) for 15 min, and observed under confocal laser scanning microscopy (CLSM, Leica SP8, Germany). Before staining, samples were divided into two groups, one group was irradiated by NIR (1 W/cm^2) for 10 min, the other group was set as control.

Real-Time Quantitative PCR Analysis: Cells were cultured on substrates in regular cell growth medium with an initial density of 1 × 10^5 cells/well. Four days later, samples were divided into two groups, one group was irradiated by NIR (1 W/cm^2) for 10 min, the other group was set as control. The total RNA was extracted using TRIzol reagent (Roche), and cDNA was generated from 1μg RNA using a Transcriptor First Strand cDNA Synthesis Kit (Roche). Real-time PCR (RT-PCR) was conducted on the LightCycler480 system (Roche) using a SYBR Green I master (Roche). Data was analyzed using the $2^{-\Delta\Delta\ CT}$ method. The relative expression of apoptosis-related genes Caspase-3 and Bcl-2 were normalized to that of the reference gene glyceraldehyde-3-phosphate dehydrogenase (GADPH). The primers for RT-PCR are listed in **Table S1**. All of the primers were purchased from Sangon Biotech.

Table S1. Primers for RT-PCR

Gene	Forward primer sequence (5'-3')	Reverse primer sequence (5'-3')
Caspase-3	AGATGGTTTGAGCCTGAGCA	CAGTGCGTATGGAGAAATGG
Bcl-2	CAACACAGACCCACCCAGA	TGGCTTCATACCACAGGTTTC

In Vivo Antitumor Activity: The experiments were approved by the Animal Care and Experiment Committee of Eastern Hepatobiliary Surgery Hospital Affiliated with The Second Military Medical University. To develop the tumor model, 4T1 cells (3×10^6) suspended in 100 μL PBS were subcutaneously injected into the back of mouse, two tumors were induced on the back of each mouse. Two weeks later, the tumor-bearing Balb/c mice were anesthetized with pentobarbital sodium (40 mg/kg) by intraperitoneal injection. Two subcutaneous pockets were made at the tumor sites and the samples were implanted under the tumors and contacted the tumors directly, the incision was then sutured. The left tumors on the back of mice were irradiated by NIR (1 W, 10 min) everyday, the right tumors on the back of mice were set as control. An infrared thermal imager was used to record the temperature change of the implanted samples under NIR irradiation, and a caliper was used to measure the tumor dimensions. The tumor volume was calculated according to the equation: Volume = Tumor length × (Tumor width)^2/2, then normalized to its initial volume (V_0) to obtain the relative tumor volume (V/V_0). An electronic balance was used to record the weight (W) of the tumor-bearing mouse, then normalized to its initial weight (W_0) to obtain the relative mice weight (W/W_0). The mice were sacrificed 12 days after sample implantation, tumors and organs (including lung, liver, spleen, kidney and heart) were dissected, fixed, dehydrated and embedded in paraffin subsequently. Histological cross-sections (~5 μm) were stained with hematoxylin-eosin (H&E). Images were obtained by a bright-field microscope (Olympus, Japan) and SEM

Data Analysis. All statistical analyses were conducted with a GraphPad Prism 5 statistical software package. All of the data were expressed as mean ± standard deviation (SD). Statistically significant differences (P) were analyzed by one-way variance and Tukey's multiple comparison tests. A value of $p < 0.05$ was considered to be statically significant, and was represented by the symbol "*", a value of $p < 0.01$ was represented by "**", and $p < 0.001$ was "***".

Sample Characterization

The formation of LDH/B starts from the pyrolysis of urea as presented in the following equations:

$$CO(NH_2)_2 \rightarrow NH_4CNO \quad (1)$$

$$NH_4CNO + H_2O \rightarrow 2NH3 + CO_2 \quad (2)$$

$$NH_3 + H_2O \leftrightarrow NH_4^+ + OH^- \quad (3)$$

So a lot of hydroxyl ions will appear and attack the nitinol substrate, leading to the activation of nitinol. The substrate start to react with hydroxyl ions in the solution, resulting the formation of NiTi-LDHs nucleus. The crystal will then start to grow with the help of nickel and titanium ions in the precursor solution, and finally form a compact NiTi-LDHs film. CO_3^{2-} ions are more easily enter into the interlayer of LDHs than butyrate, so the interlayer anions in the primary formed LDHs are mainly composed by CO_3^{2-}. However, the concentration of butyrate (300 mM) in the precursor solution is higher than urea (about 100 mM), and only a small part of urea can totally pyrolysis to produce CO_3^{2-}. So the concentration of butyrate is much higher than carbonate, and butyrate will exchange with the interlayer carbonate, leading to the formation of LDH/B.

XRD spectra of different samples were presented in **Figure S1**. A strong reflection peak centered approximately 11°, the most characteristic peak corresponding to the (0 0 3) crystal face of NiTi-LDHs, was detected in LDH, LDH/B, Au@LDH and Au@LDH/B samples. The (0 0 3) peak in LDH and Au@LDH centered at 11.46°, but changed to 11.29° in LDH/B and

Au@LDH/B samples, indicating that the basal spacing of NiTi-LDHs changed from 0.762 nm to 0.783 nm. After GNRs loading, peaks centered approximately 38° and 44° appeared, corresponding to the (1 1 1) and (2 0 0) crystal face of metallic gold (PDF#99-0056).

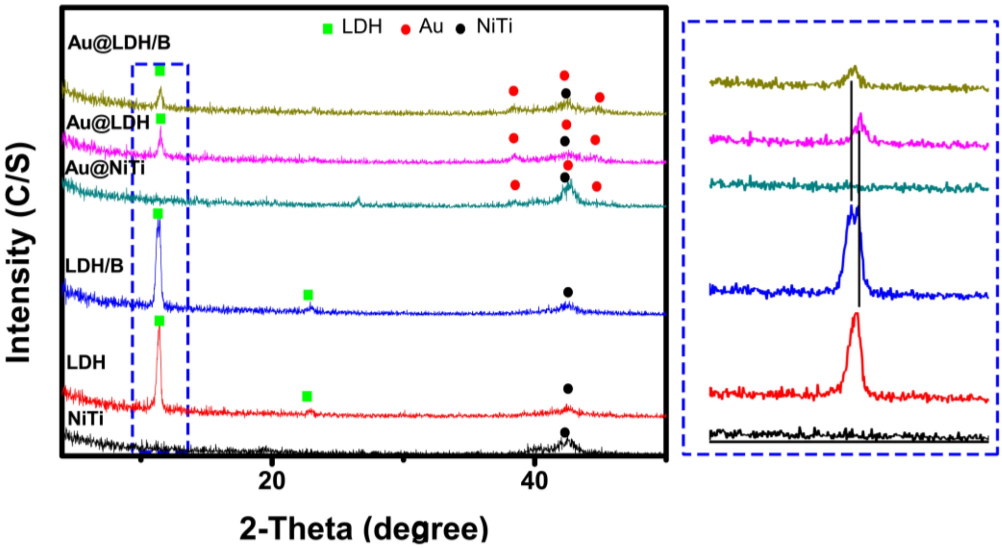

Figure S1 XRD spectra of different samples.

Fourier transform infrared spectroscopy (FTIR) tests were conducted to characterize the interlayer anions (**Figure S2**). A strong peak centered at 2225 cm^{-1} was assigned to the C-N stretching of the cyanate anion (CNO^-), which resulted from the incomplete decomposition of urea.[1] Peaks corresponding to the vibration of carbonate (CO_3^{2-}) could be detected approximately 1384 cm^{-1}, in the pattern of LDH sample, indicating the coexistence of CNO^- and CO_3^{2-} in the LDH gallery.[2] In the case of LDH/B, two sharp bands at 1405 and 1558 cm^{-1} corresponding to the symmetry and asymmetry vibration of C=O in carboxylate could be detected, while bands approximately 2939 and 2964 cm^{-1} could be assigned to $\nu_{CH2(as)}$ and $\nu_{CH3(as)}$, respectively.[3] These results demonstrated that butyrate ions had been successfully inserted into the interlayer of the prepared LDH/B. After GNRs loading, peaks corresponding to CNO^- disappeared, owning to the interlayer ions release in the GNRs loading process. However, peaks corresponding to carbonate and butyrate could be still detected in Au@LDH and Au@LDH/B respectively, indicating their interlayer carbonate and butyrate would not be affected by GNRs loading.

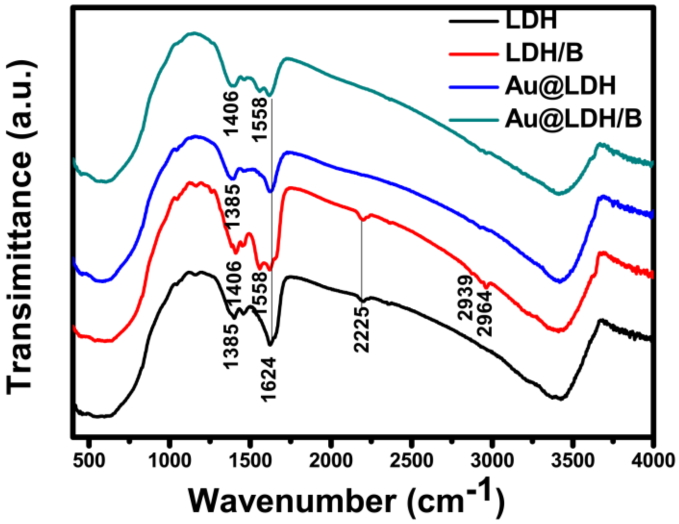

Figure S2 FTIR spectra of different samples.

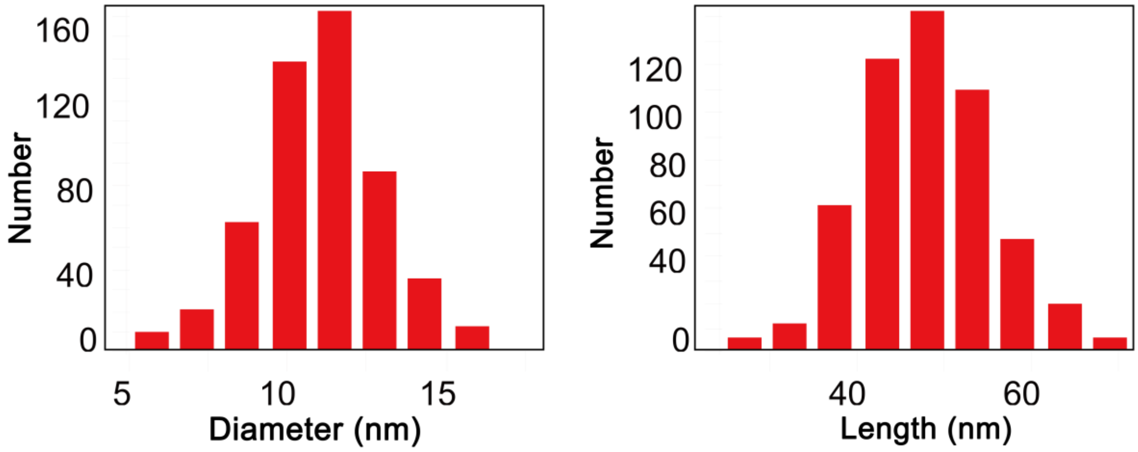

Figure S3 Histogram showing the size distribution of diameter and length of GNRs.

XPS spectra of different samples were presented in **Figure S4**, the corresponding quantification data was presented in **Table S2**. Only Ni, Ti, O, C could be detected in LDH and LDH/B samples. After GNRs loading, peaks corresponding to Au appeared, and C content increased, owning to C is the major component of PSS and APS, which were used in the immobilization of GNRs on NiTi-LDHs films.

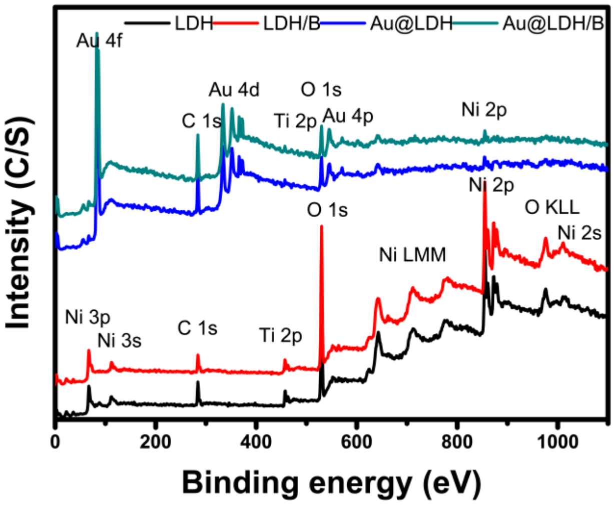

Figure S4 XPS spectra of different samples.

Table S2 Elemental compositions of different samples.

	C (at. %)	O (at. %)	Ni (at. %)	Ti (at. %)	Au (at. %)
LDH	25.32	62.46	8.96.	3.26	0
LDH/B	24.05	64.38	8.59	2.98	0
Au@LDH	68.51	18.33	1.53	1.41	10.21
Au@LDH/B	66.88	18.70	1.24	0.87	12.32

Ti 2p, Ni 2p and Au 4f XPS spectra of different samples were presented in **Figure S5**. Ti 2p XPS spectra presented two peaks centered at 458.2 eV (Ti $2p_{3/2}$) and 464.0 eV (Ti $2p_{1/2}$) due to the presence of Ti^{4+}. It should be noted that the tested value is slightly lower than the reported value of Ti^{4+} in TiO_2.[4] The binding energy of a metal is determined by the positive potential that results from the specific arrangement of the ion core and electrons. The decrease in the binding energy of Ti $2p_{3/2}$ and $2p_{1/2}$ for the NiTi-LDHs materials is due to a weak bonding situation from the introduction of Ni relative to the Ti-O-Ti bonding in TiO_2.[1] Besides two shakeup satellites (indicated as "Sat"), two major peaks at 855.8 and 873.4 eV could be detected in Ni 2p XPS spectra, corresponding to Ni $2p_{3/2}$ and Ni $2p_{1/2}$, respectively. The spin-energy separation of 17.6 eV is characteristic of Ni^{2+} in $Ni(OH)_2$.[5] After GNRs loading, Au 4f peaks appeared in the high resolution XPS spectra. Two peaks at 83.5 and 87.2 eV could be detected in both of Au@LDH and Au@LDH/B samples, corresponding to Au $4f_{7/2}$ and Au $4f_{5/2}$. The spin-energy separation of 3.7 eV is characteristic of Au^0,[6] confirming the loaded GNRs were composed of metallic gold.

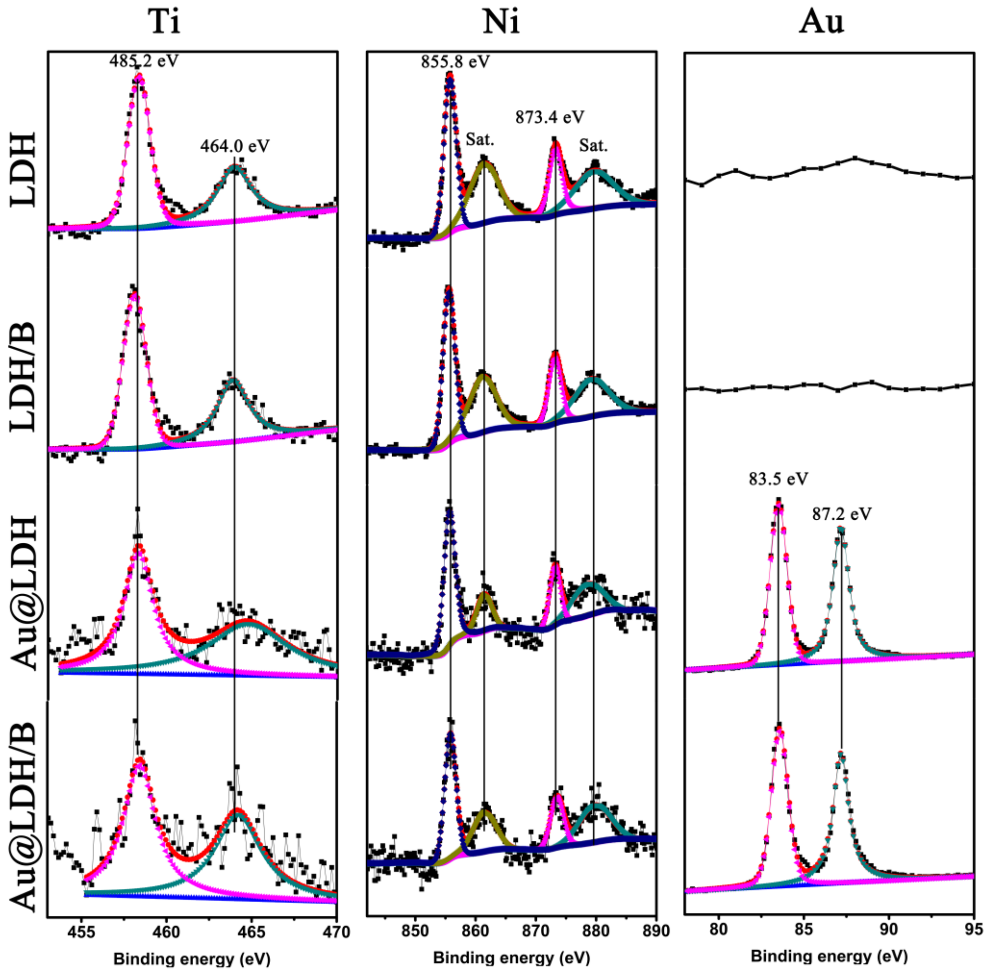

Figure S5 High resolution XPS spectra of Ti 2p, Ni 2p and Au 4f.

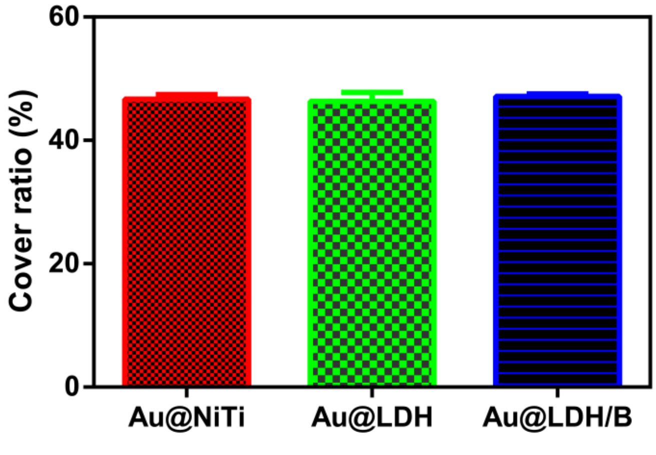

Figure S6 GNRs cover ratio of Au@NiTi, Au@LDH and Au@LDH/B samples.

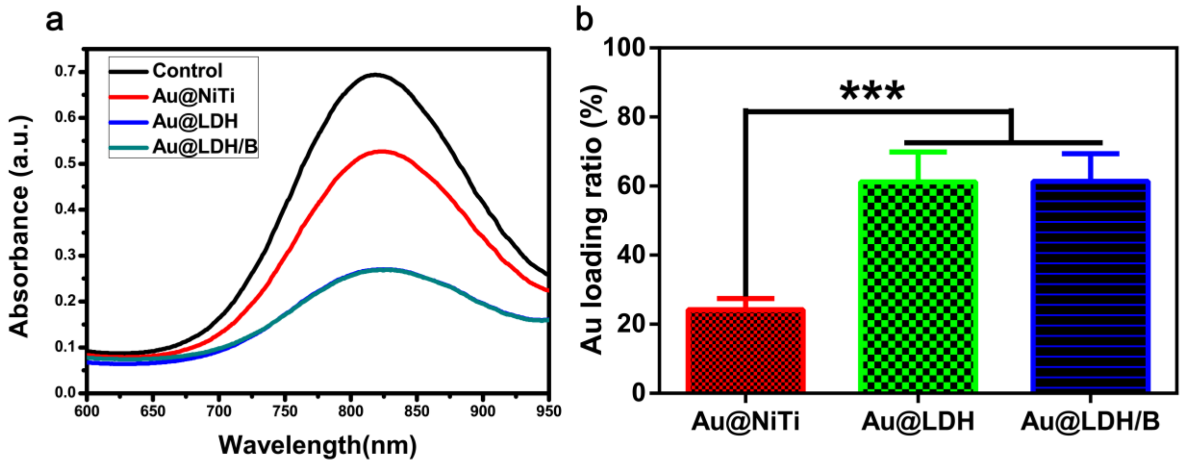

Figure S7 (a) UV-Vis spectra of the GNRs sol tested before and after immersed by different samples, (b) GNRs loading ratio of different samples.

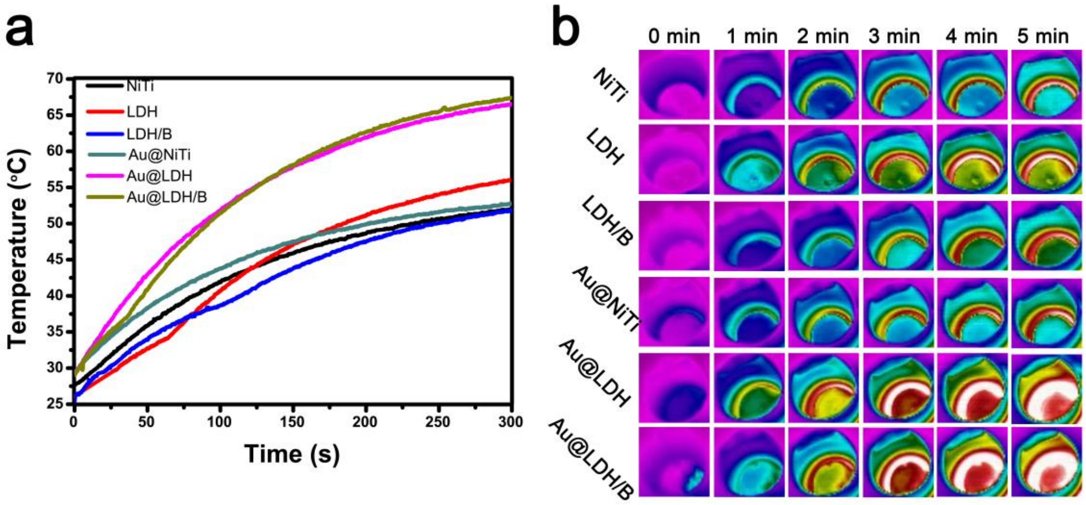

Figure S8 (a) The changes of surface temperature of different samples in air under NIR irradiation; (b) images showing the temperature distribution.

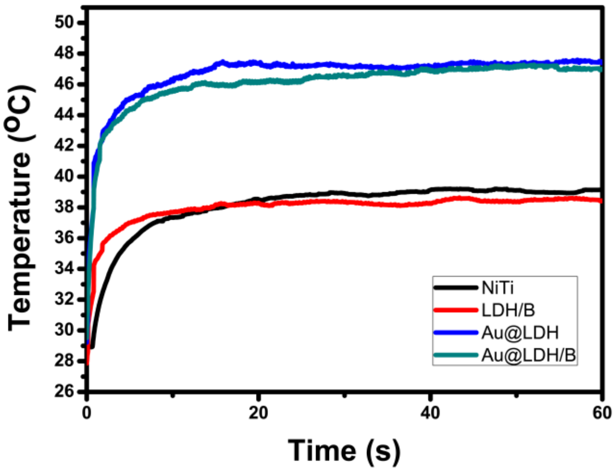

Figure S9 The changes of surface temperature of different samples implanted *in vivo*.

Calculation of Photothermal Conversion Efficiency

The photothermal conversion efficiency of the GNRs modified NiTi-LDHs film was calculated by a modified method similarly to Korgel's report.[7] Sample with an area of 1 cm^2 was placed into 24-well plate containing 500 μL ultrapure water, the total energy balance for the system can be expressed by **Equation S4**:

$$\sum m_i C_{P,i} \frac{dT}{dt} = Q_{Au} + Q_{Dis} - Q_{in,Surr} \quad (4)$$

where m and c_p are the mass and heat capacity of water, respectively, T is the solution temperature, Q_{Au} is the energy inputted by GNRs, Q_{Dis} is the baseline energy inputted by the sample cell, and $Q_{in,Surr}$ is the heat conduction away from the system by air.

The laser-induced source term Q_{Au}, represents heat inputted by the photothermal conversion effect of GNRs immobilized on the NiTi-LDHs film under the irradiation of 808 nm laser, and can be expressed by **Equation S5**:

$$Q_{Au} = I\left(1 - 10^{-A(808)}\right)\eta \quad (5)$$

where I is the laser power, η is the photothermal conversion efficiency of the GNRs-modified NiTi-LDHs films, $A_{(808)}$ is the absorbance of sample at wavelength of 808 nm (**Figure S10**).

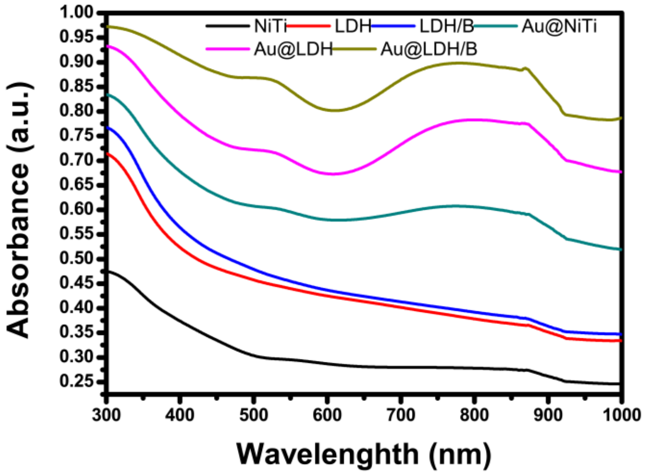

Figure S10 UV-Vis spectra of different samples.

Q_{Dis} represents heat dissipated from light absorbed by the NiTi-LDHs substrates and solvent, which was measured independently and found to be 36.5 mW.

$Q_{in,Surr}$ is linear with temperature, as given by **Equation S6**:

$$Q_{in,Surr} = hA\left(T - T_{Surr}\right) \quad (6)$$

Where h is heat transfer coefficient, A is the surface area of the container, and T_{Surr} is the temperature of the surroundings.

At the maximum steady-state temperature (T_{Max}), the rate of photothermal heating is then equal to the rate of heat transfer out of the system:

$$Q_{Au} + Q_{Dis} = hA\left(T_{Max} - T_{Surr}\right) \quad (7)$$

Substituting **Equation S5** and **S6** into **Equation S7** and rearranging to get:

$$\eta = \frac{hA\left(T_{Max} - T_{Surr}\right) - Q_{Dis}}{I\left(1 - 10^{-A(808)}\right)} \quad (8)$$

where (T_{Max} - T_{Surr}) is 25.2 °C (**Figure S11**a), Q_{Dis} is 36.5 mW, thus only hA remains unknown for calculating η. In order to get hA, a dimensionless driving force temperature θ is introduced:

$$\theta = \frac{T - T_{Surr}}{T_{Max} - T_{Surr}} \quad (9)$$

and a sample system time constant τ_S:

$$\tau_S = \frac{\Sigma m_i C_{p,i}}{hA} \quad (10)$$

which are substituted into **Equation S3** and rearranged to yield:

$$\frac{d\theta}{dt} = \frac{1}{\tau_s}\left[\frac{Q_{Au} + Q_{Dis}}{hA(T_{Max} - T_{Surr})} - \theta\right] \quad (11)$$

At the cooling state, the laser source was shut off, the $Q_{Au} + Q_{Dis} = 0$, reducing the **Equation S12:**

$$dt = -\tau_s \frac{d\theta}{\theta} \quad (12)$$

And integrating, giving the expression:

$$t = -\tau_s \ln\theta \quad (13)$$

Therefore, time constant for heat transfer from the system is determined by applying the linear time data from the cooling period *vs* negative natural logarithm of driving force temperature (**Figure S11**b). Thus, according to **Equation S10**, the *hA* is deduced to be 7.3 mW/°C. Substituting 7.3 mW/°C of the *hA* into **Equation S8**, the 808 nm laser heat conversion efficiency (η) of Au@LDH/B can be calculated to be 21.7%.

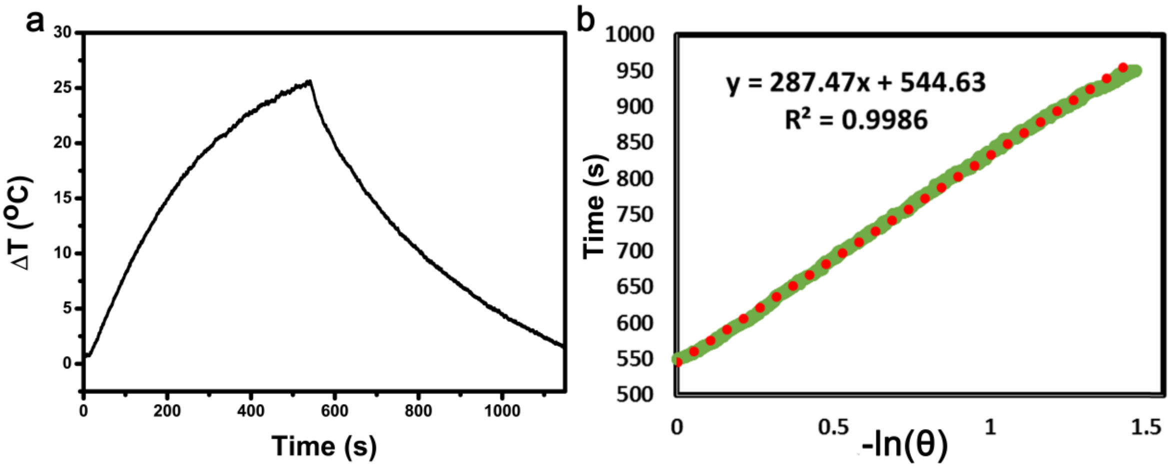

Figure S11 (a) Temperature variation of sample Au@LDH/B with and without near infrared irradiation; (b) the linear relationship between the negative logarithm of θ and time in the cooling stage.

Calculation of the Temperature Distribution of GNRs Modified on NiTi-LDHs Film

To obtain the temperature distribution of GNRs, their convective heat transfer coefficient (h_w) in water should be calculated first. GNRs are reducing to columns with a diameter (D) of 10 nm, and length (L) of 50 nm. The Grashof number (Gr) of GNRs can be expressed by **Equation S14**:

$$(Gr \bullet \Pr)_b = \frac{g\beta\Delta T D^3 \Pr}{v^2} \quad (14)$$

Where g is the gravitational acceleration, β is the coefficient of cubic expansion of water, ΔT is the temperature differences between sample and environment, υ is the viscosity of water, Pr is the Prandtl number. Based on the testing results showing in **Figure 1**e, the quantitative temperature is set to be 50 °C, and the Gr is calculated to be 2.02×10^{-13}, indicating that the

fluid flow state is consistent with the elongated cylinder-type. Therefore, the Nuselt number of GNRs can be expressed by **Equation S15**:[8]

$$Nu = 0.95\left(Gr\ \Pr\ \frac{D}{L}\right)^{0.052} \tag{15}$$

the Nuselt number can be also expressed as：

$$Nu = \frac{h_w D}{\lambda} \tag{16}$$

Where λ is the conductivity coefficient of water. The convective heat transfer coefficient (h_w) of GNRs can be obtained by solving the above two simultaneous equations (**Equation S15** and **S16**). The result is $h_w = 1.2 \times 10^7$ W/m^2•°C.

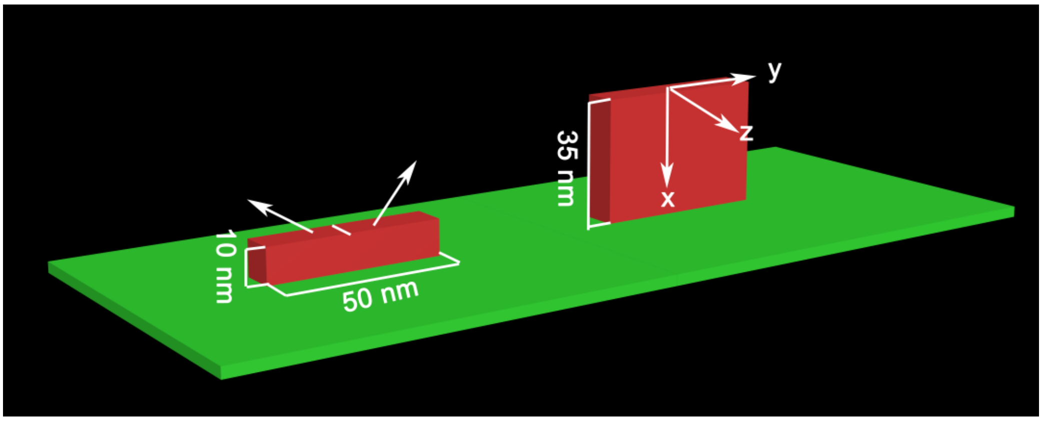

Figure S12 Simplified model for calculation.

To simplify the calculation, the shape of GNRs is simplified to a 10 nm × 10 nm × 50 nm regular square prism. To create adiabatic condition, the top surface of prim is unfolded, forming a new prism with dimensions of 10 nm × 35 nm × 50 nm. Establish rectangular coordinate system with the center of the top surface of the newly formed prism as its origin (**Figure S12**), the thermal conduction differential equation of the system can be expressed by **Equation S17**:

$$\frac{\partial^2 T}{\partial x^2} + \frac{\partial^2 T}{\partial y^2} + \frac{\partial^2 T}{\partial z^2} + \frac{\phi}{\lambda_{Au}} = 0 \tag{17}$$

the above formula can be reduced to **Equation S18**:

$$\frac{d^2 T}{dx^2} + \frac{\phi}{\lambda_{Au}} = 0 \tag{18}$$

Where λ_{Au} is the heat conductivity coefficient of GNRs; Φ is the total heat induced into the system, and can be expressed by **Equation S19**:

$$\phi = \frac{\left[q - h_w\left(T - T_s\right)\right]\left(L_{\mathrm{peri}}dx\right)}{A_c} \tag{19}$$

where q is the heat induced by GNRs under NIR irradiation, T_s is the temperature of water, L_{peri} is the perimeter of the cross section of GNRs, A_c is the area of the cross section.

To solve **Equation S18**, parameter χ is induced:

$$\chi = T - T_S - \frac{q}{h_w} \tag{20}$$

substitute **Equation S19** and **S20** into **Equation S18** and rearrange to yield:

$$\frac{d^2\chi}{dx^2} = \frac{hL_{Peri}}{\lambda_{Au}A_c}\chi \tag{21}$$

induce parameter *m*:

$$m = \sqrt{\frac{hL_{Peri}}{\lambda_{Au}A_c}} \tag{22}$$

which is substituted into **Equation S21** and rearranged to yield a general analytic solution:

$$\chi = c_1e^{mx} + c_2e^{-mx} \tag{23}$$

boundary conditions are substituted into **Equation S23** to yield:

$$\chi = \chi_0\frac{e^{mx} + e^{2mH}e^{-mx}}{1 + e^{2mH}} = \chi_0\frac{ch[m(x - H)]}{ch(mH)} \tag{24}$$

after the exact figures are substituted into **Equation S24**, the temperature distribution of GNRs modified on NiTi-LDHs film can be obtained:

$$T = (-40113.1\cosh(3014162.2x - 0.105) + 40384.5)^{\circ}C \qquad 0 \le x \le 35 \text{ nm} \tag{25}$$

The corresponding temperature distribution curve is shown in **Figure 4**a.

NIR-Induced Crystal Phase Transformation

The thermogravimetric (TG) and differential scanning calorimeter (DSC) curves of LDH/B sample is shown in **Figure S13**, endothermic peak centered 100 °C and 180 °C corresponding to the desorption of absorbed water and lattice water respectively. Exothermic peak centered approximately 350 °C is assigned to the crystal phase change of NiTi-LDHs. The transformation onset temperature is obtained to be 254 °C via a tangent method.

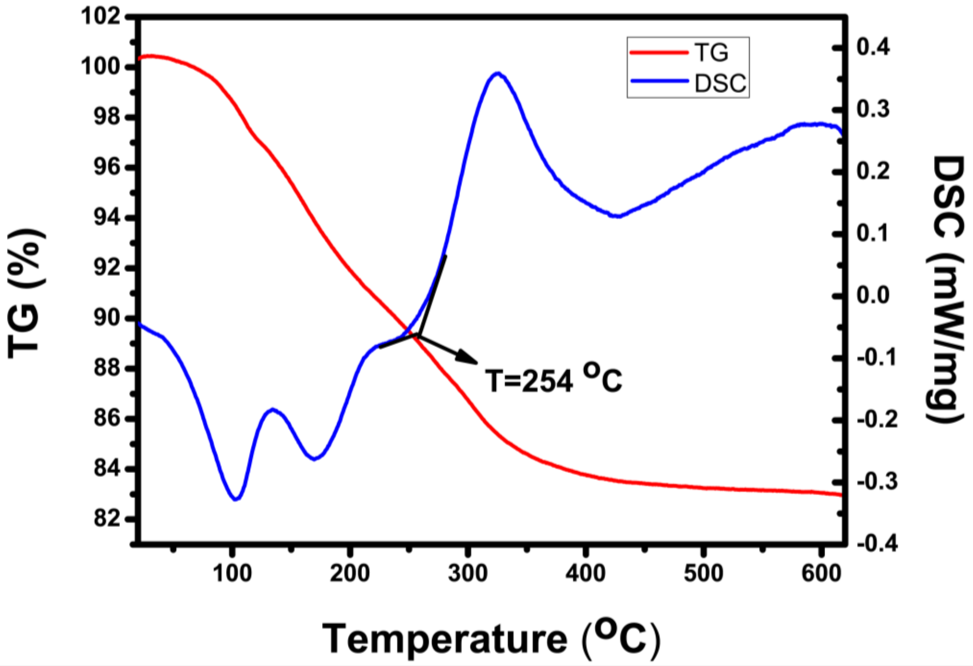

Figure S13 TG and DSC curves of LDH/B sample.

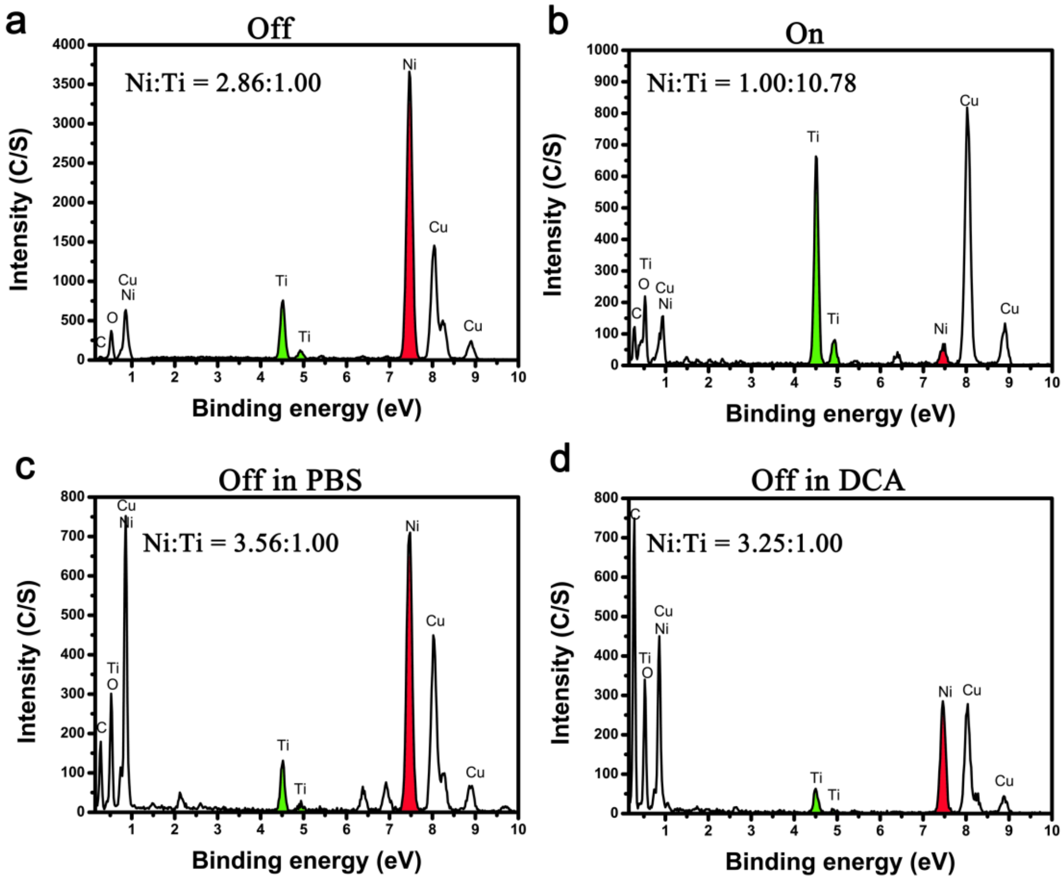

Figure S14 EDS spectra corresponding to the area showed in the red square draw in **Figure 2**c.

Before NIR irradiation, the Ni/Ti ratio in the area near GNRs was 2.86:1, consisting with the initial ratio of the cations in the precursor solution (3:1). After NIR irradiation, the Ni/Ti ratio changed to 1.00:10.78, confirming the phase transformation. Immersing the NIR-irradiated Au@LDH/B into PBS or DCA solution, its Ni/Ti recovered to approximately 3:1, indicating the reverse transformation of NiTi-LDOs to NiTi-LDHs.

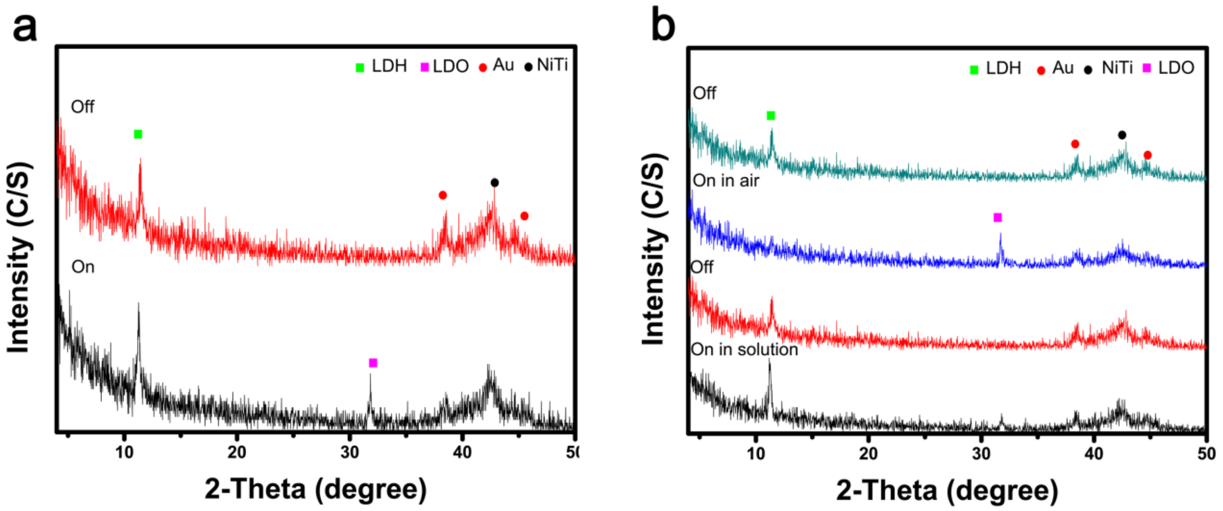

Figure S15 (a) Influence of the NIR irradiation to the crystalline phase of Au@LDH/B sample immersed in water; (b) influence of the NIR irradiation to the crystalline phase of Au@LDH/B sample after 20 cycles of crystalline phase transformation.

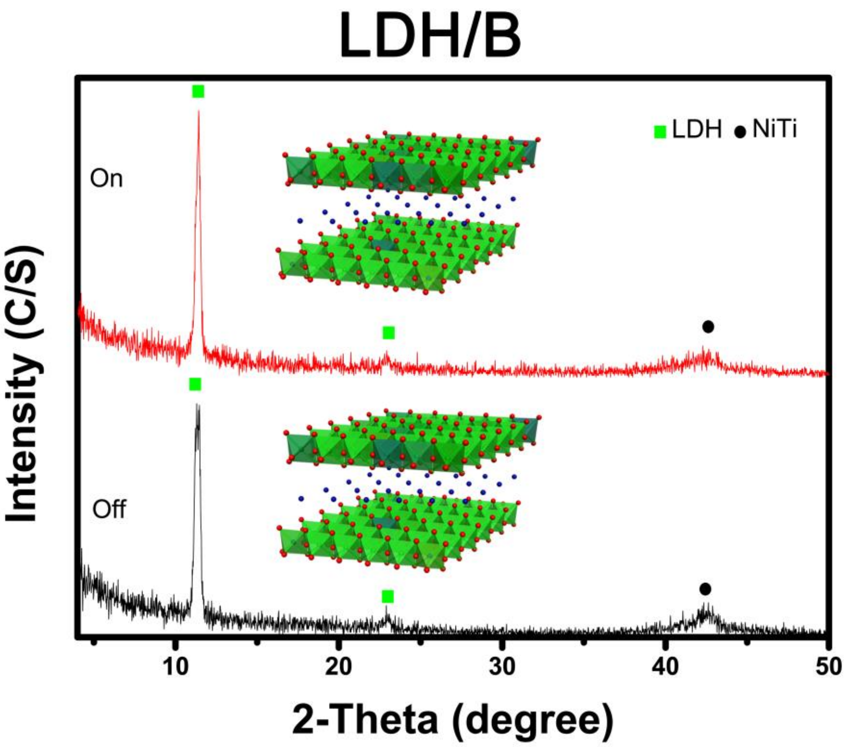

Figure S16 Influence of the NIR irradiation to the crystalline phase of LDH/B sample.

In Vitro Cell Experiments Results

In vitro cell experiments verified that the prepared films had little adverse effect to normal cells, but can effectively kill cancer cells under NIR irradiation.

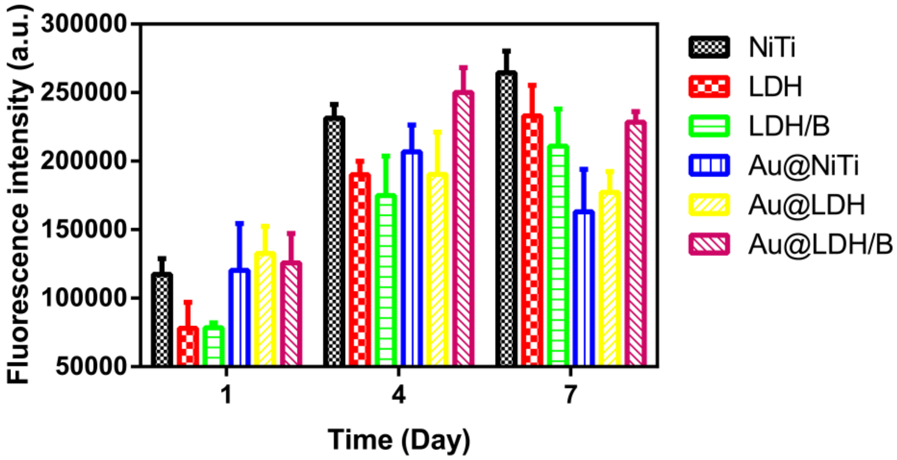

Figure S17 Proliferation of normal cells cultured on different samples

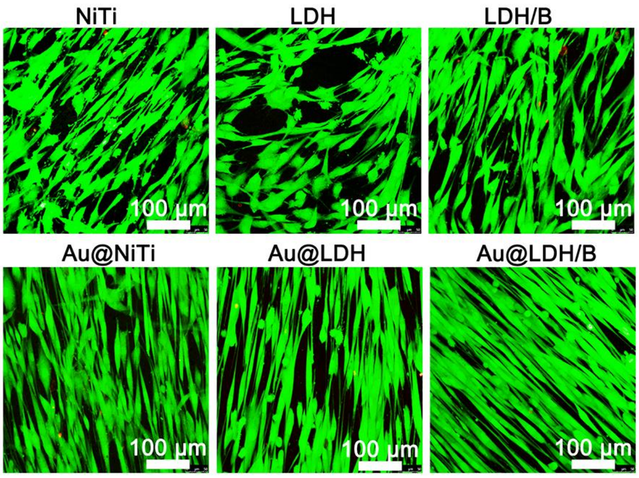

Figure S18 CLSM images of live/dead stained HIBEpiC cells cultured on different samples.

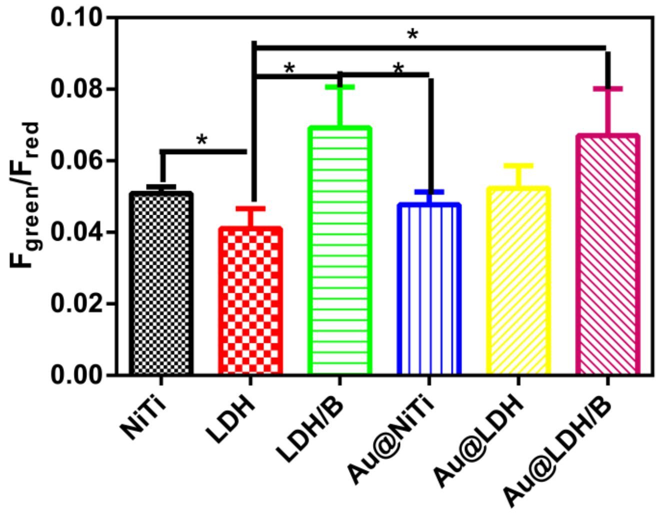

Figure S19 Early apoptosis of HIBEpiC cells cultured on different samples.

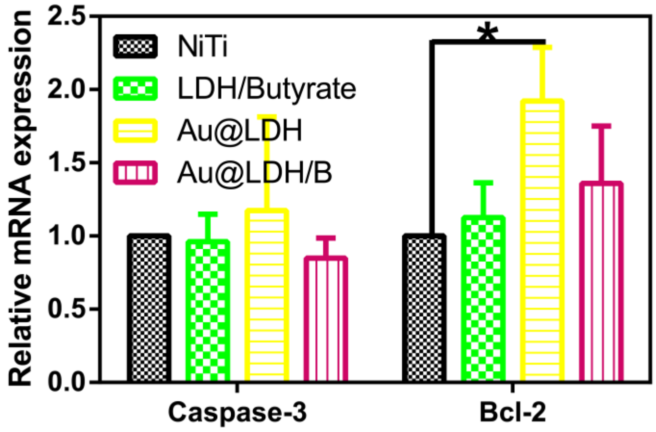

Figure S20 The relative expression of the apoptosis related genes Caspase-3 and Bcl-2 in HIBEpiC cells cultured on different samples.

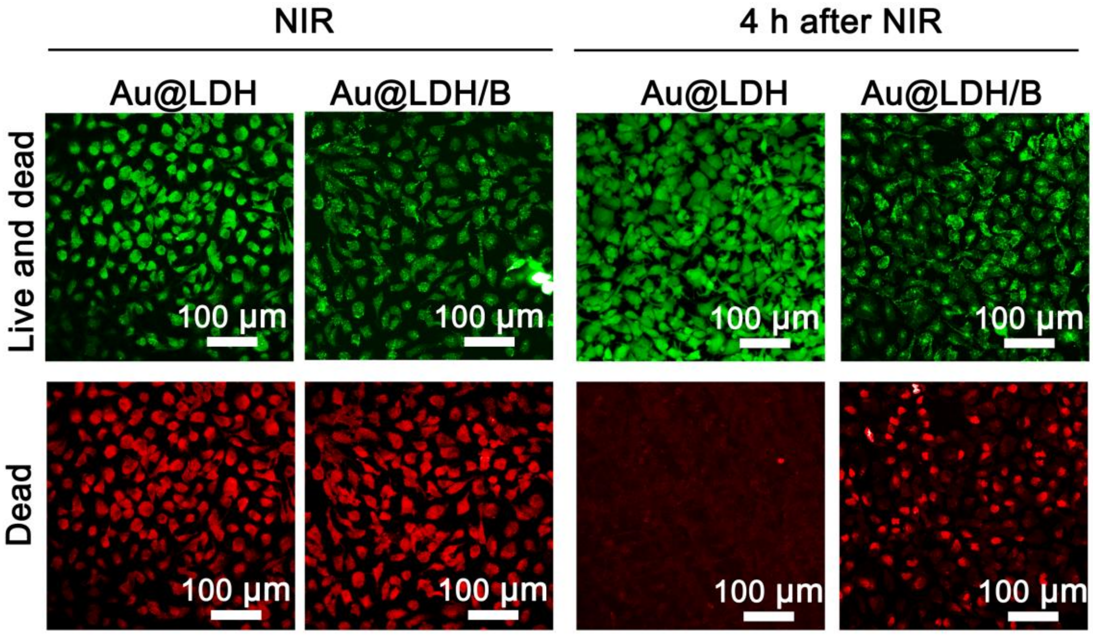

Figure S21 CLSM images of the live/dead stained RBE cells cultured on Au@LDH and Au@LDH/B 0 h and 4 h after the NIR irradiation.

High temperature stimulation will make cells come to an autophagy state[1]. In this state, the cell membranes are broken to some extent, so the PI molecule can enter into the inside of the cells and bind with the DNA molecule to emit red fluorescence; as the nucleus are also destroyed, chromosomes will disperse into the cytoplasm, making the entire cell was red stained (Fig. S21 in the Supporting Information). However, after stopping the NIR irradiation, the temperature return to the normal level, cells thus recover from autophagy state to a normal state, making cell on Au@LDH turned from red to green. On the contrary, because of the butyrate release from Au@LDH/B under NIR irradiation, cells on Au@LDH/B were still in an inhibited state even after the stopping of NIR irradiation, and eventually killed by the released butyrate, making PI thoroughly enter into the nucleus. The above results are consistent with the quantification results shown in Fig. 6a, b, verifying that applying NIR irradiation alone cannot kill cancer cells.

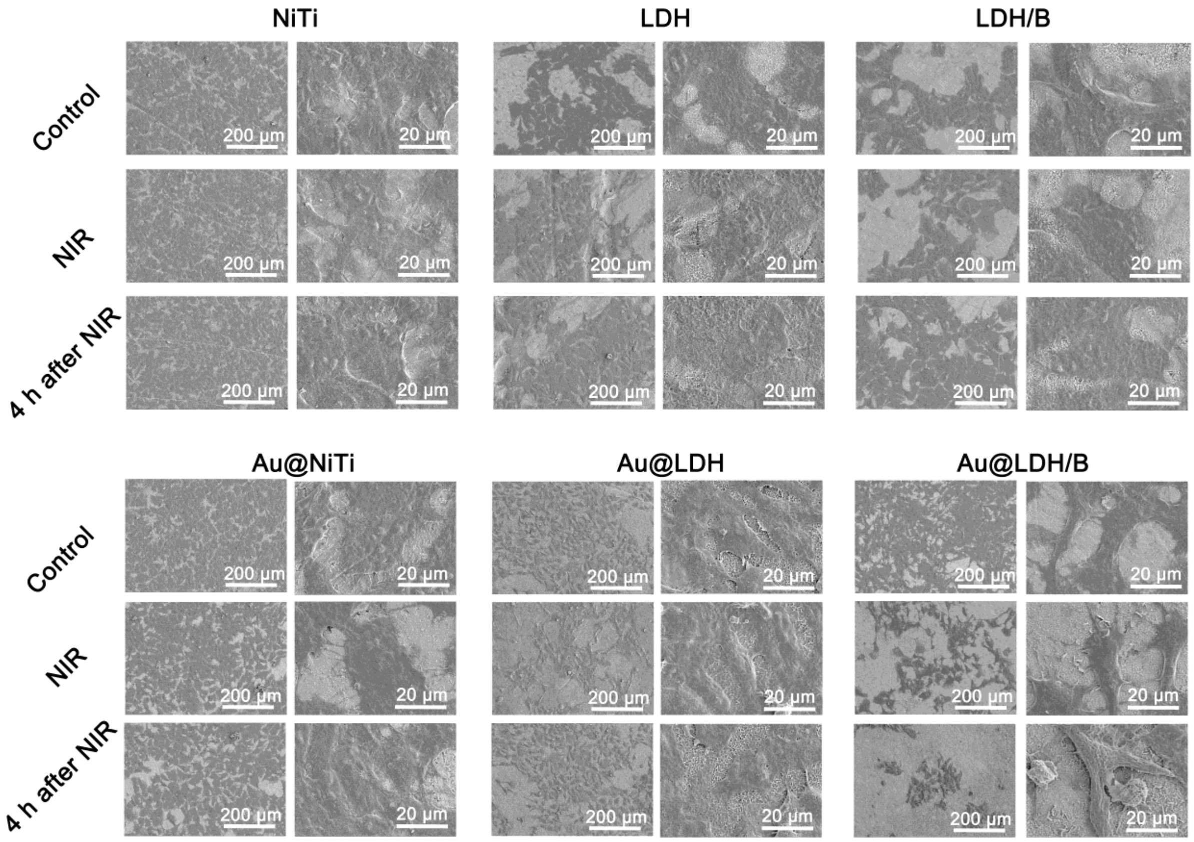

Figure S22 SEM images of RBE cells cultured on different samples before and after the NIR irradiation.

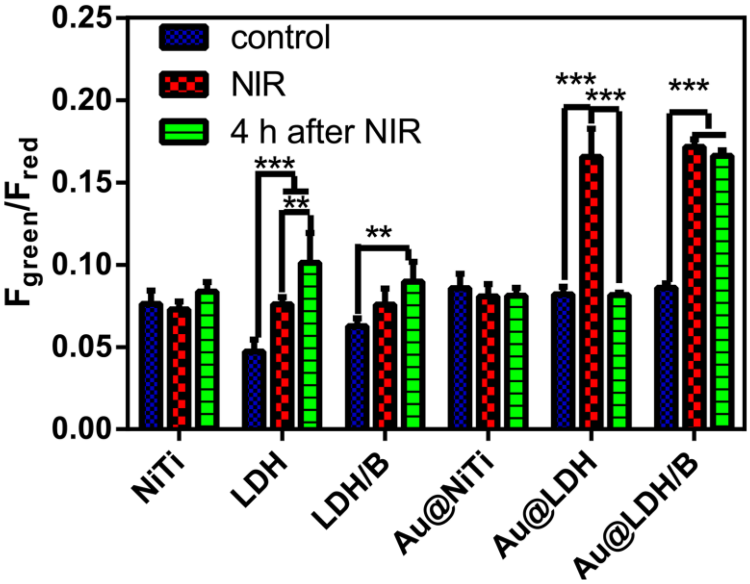

Figure S23 Early apoptosis of cancer cells cultured on different samples before (control), just after and 4 h after NIR irradiation.

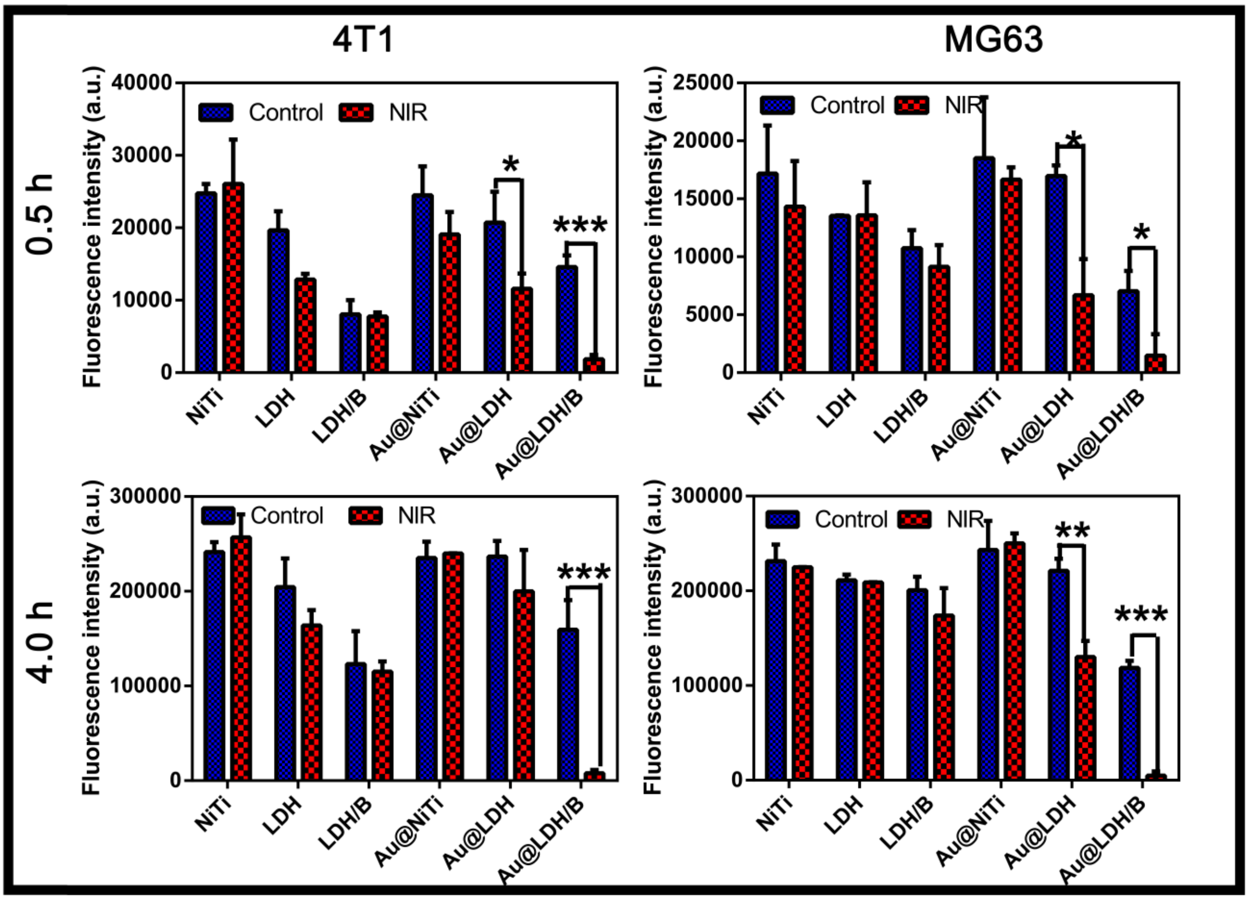

Figure S24 Cell viability of 4T1 and MG63 cells cultured on different samples before and after the NIR irradiation.

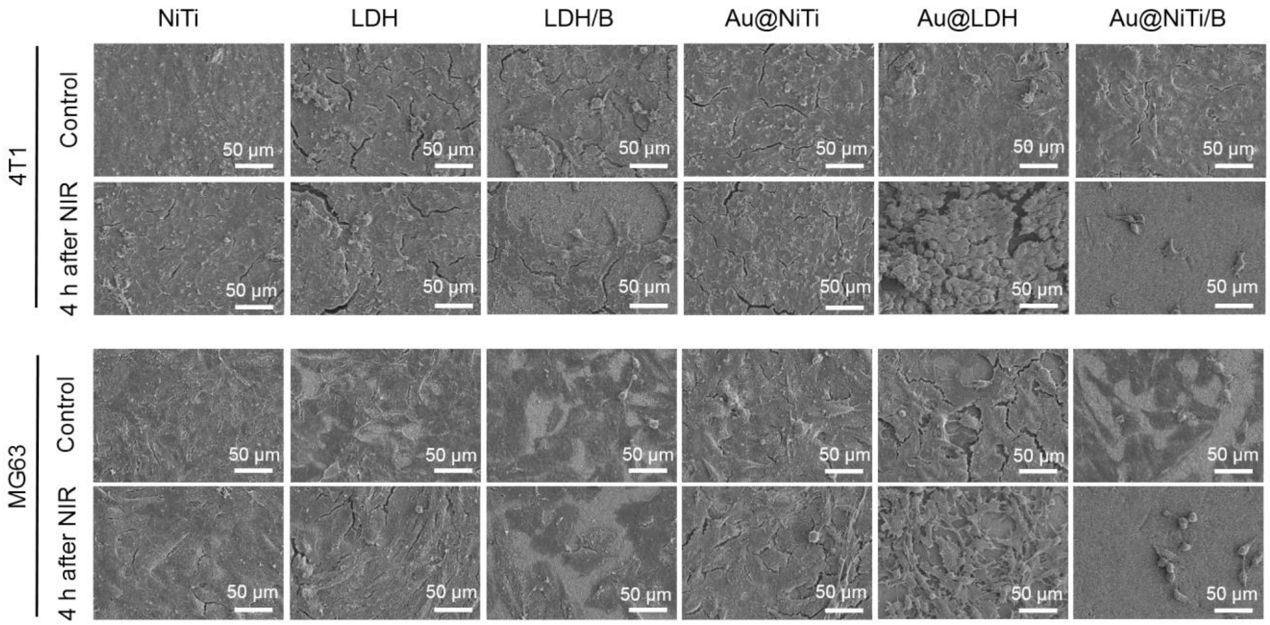

Figure S25 SEM images of 4T1 and MG63 cells cultured on different samples before and after the NIR irradiation.

***In Vivo* Experiments Results**

In vivo experiments further verified the high biocompatibility and tumor inhibition efficiency of the prepared films under NIR irradiation.

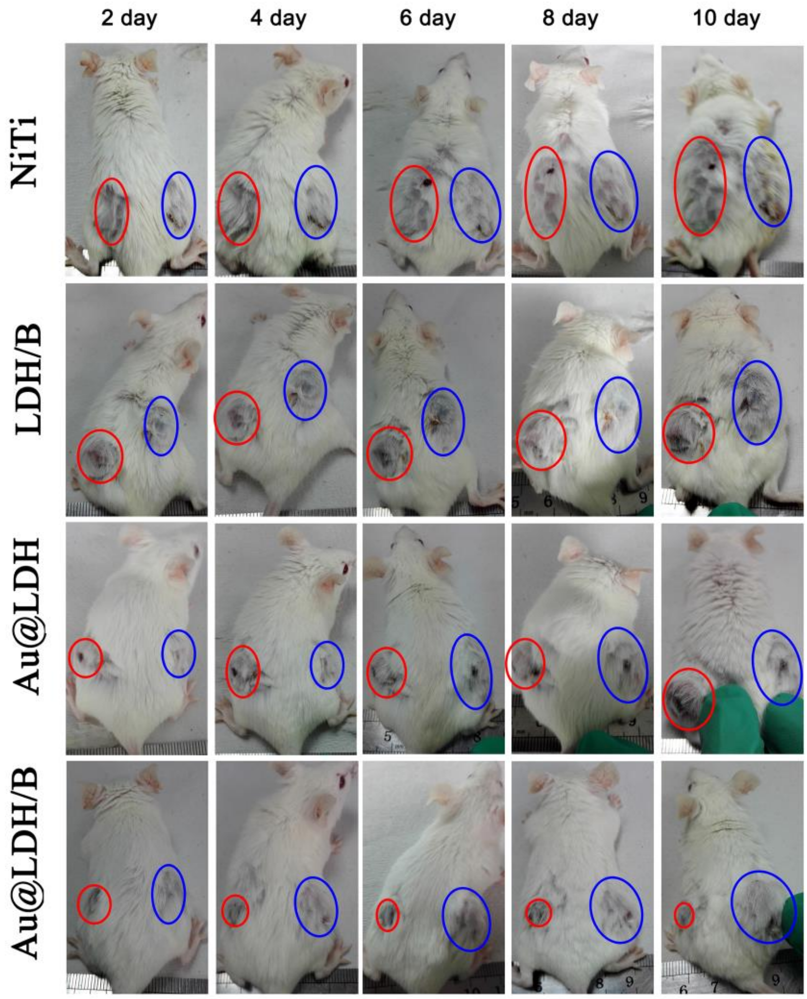

Figure S26 Representative digital images of tumor-bearing mice after being implanted with different samples for various time periods, the left tumor was irradiated by NIR (red circle), the right tumor was set as control (blue circle).

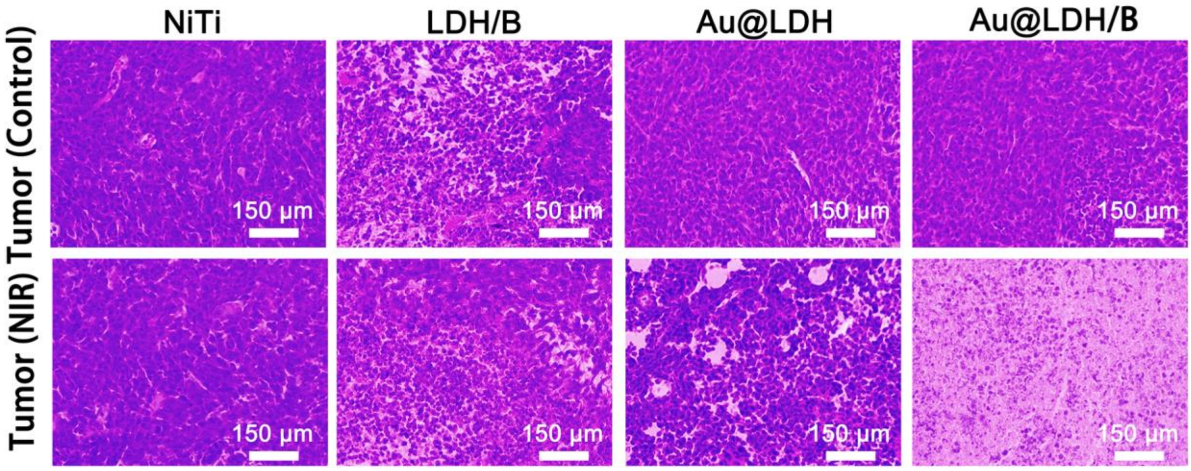

Figure S27 Histological changes in tumor tissues contacting different samples without (Control group) and with (NIR group) NIR irradiation.

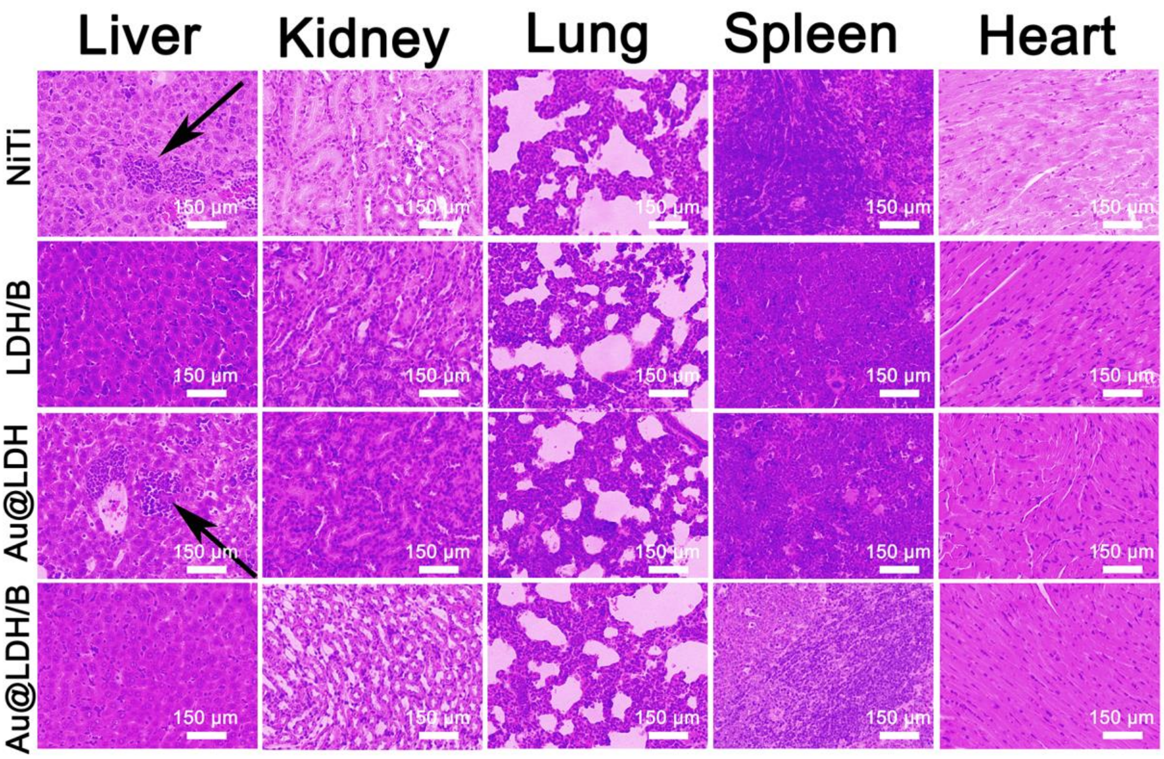

Figure S28 Histological changes in various tissues of tumor-bearing mice implanted with different samples.

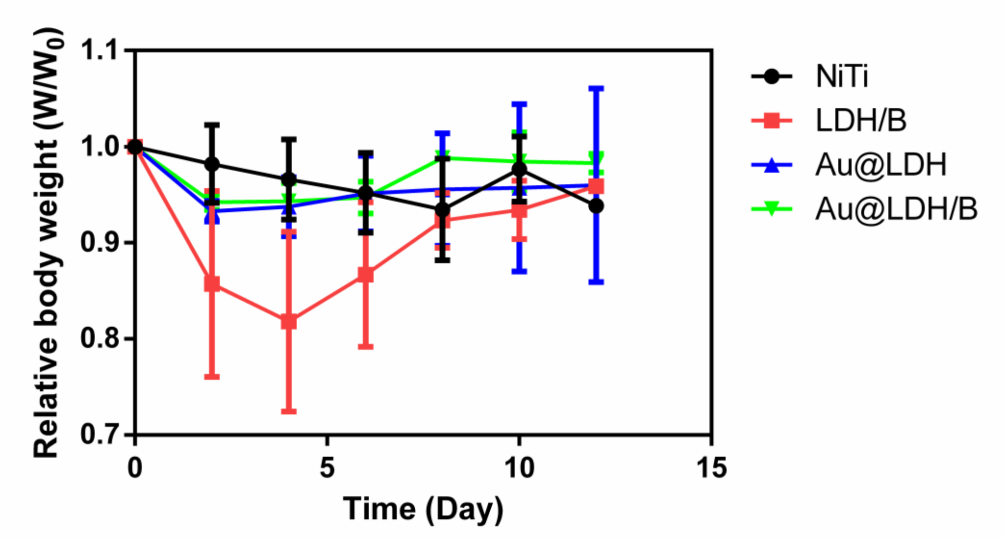

Figure S29 Relative body weight of the tumor-bearing mice after being implanted with different samples for various time periods

Supporting Movies

Movie 1 Representative video recording of tumor-bearing mouse after being implanted with NiTi sample for 10 days.

Movie 2 Representative video recording of tumor-bearing mouse after being implanted with LDH/B sample for 10 days.

Movie 3 Representative video recording of tumor-bearing mouse after being implanted with Au@LDH sample for 10 days.

Movie 4 Representative video recording of tumor-bearing mouse after being implanted with Au@LDH/B sample for 10 days.

Reference

[1] Y. Zhao, P. Chen, B. Zhang, D. S. Su, S. Zhang, L. Tian, J. Lu, Z. Li, X. Cao, B. Wang, M. Wei, D. G. Evans, X. Duan, Chemistry 2012, 18, 11949.
[2] T. Zhang, Q. Li, H. Xiao, H. Lu, Y. Zhou, Industrial & Engineering Chemistry Research 2012, 51, 11490.
[3] G. Kavitha, C. Kurinjimalar, K. Sivakumar, R. Aravind, C. G. Shree, K. Arthi, P. Palani, V. Kaviyarasan, R. Rengasamy, International Journal of Biological Macromolecules 2016, 93, 1304.
[4] I. T. Chashechnikova, V. M. Vorotyntsev, V. V. Borovik, G. I. Golodets, I. V. Plyuto, A. P. Shpak, Teoreticheskaya I Eksperimentalnaya Khimiya 1992, 28, 216; H. Noda, K. Oikawa, T. Ogata, K. Matsuki, H. Kamada, Nippon Kagaku Kaishi 1986, 1084.
[5] H. Wang, X. Xiang, F. Li, Journal of Materials Chemistry 2010, 20, 3944; X. Cai, X. Shen, L. Ma, Z. Ji, C. Xu, A. Yuan, Chemical Engineering Journal 2015, 268, 251.
[6] J. Hedman, R. Nilsson, S. A. Nemnonov, C. Nordling, V. A. Trapeznikov, M. F. Sorokina, O. I. Kljushnikov, M. Klasson, Physica Scripta 1971, 4, 195; T. D. Thomas, P. Weightman, Physical Review B 1986, 33, 5406.
[7] D. K. Roper, W. Ahn, M. Hoepfner, Journal of Physical Chemistry C 2007, 111, 3636.
[8] W. Tao, *Heat transfer theory*, Northwestern Polytechnic University Press, Xi'an 2006.
[1] aW. Y. Xie, X. D. Zhou, J. Yang, L. X. Chen, D. H. Ran, *Arch. Biochem. Biophys.* **2016**, *607*, 55-66; bG. Stirnemann, F. Sterpone, *The journal of physical chemistry letters* **2017**, 5884-5890.
